# Lysophosphatidic acid receptor LPA_3_ prevents oxidative stress and cellular senescence in Hutchinson–Gilford progeria syndrome

**DOI:** 10.1111/acel.13064

**Published:** 2019-11-12

**Authors:** Wei‐Min Chen, Jui‐Chung Chiang, Yueh‐Chien Lin, Yu‐Nung Lin, Pei‐Yun Chuang, Ya‐Chi Chang, Chien‐Chin Chen, Kao‐Yi Wu, Jung‐Chien Hsieh, Shih‐Kuo Chen, Wei‐Pang Huang, Benjamin P. C. Chen, Hsinyu Lee

**Affiliations:** ^1^ Department of Life Science National Taiwan University Taipei Taiwan; ^2^ Department of Radiation Oncology University of Texas Southwestern Medical Center Dallas TX USA; ^3^ Department of Pathology Ditmanson Medical Foundation Chia‐Yi Christian Hospital Chiayi Taiwan; ^4^ Department of Cosmetic Science Chia Nan University of Pharmacy and Science Tainan Taiwan; ^5^ Department of Electrical Engineering National Taiwan University Taipei Taiwan; ^6^ Institute of Biomedical Electronics and Bioinformatics National Taiwan University Taipei Taiwan; ^7^ Center for Biotechnology National Taiwan University Taipei Taiwan

**Keywords:** 1‐Oleoyl‐2‐O‐methyl‐rac‐glycerophosphothionate, cell senescence, Hutchinson–Gilford progeria syndrome, LPA_3_, lysophosphatidic acid, reactive oxygen species

## Abstract

Hutchinson–Gilford progeria syndrome (HGPS) is a rare laminopathy that produces a mutant form of prelamin A, known as Progerin, resulting in premature aging. HGPS cells show morphological abnormalities of the nuclear membrane, reduced cell proliferation rates, accumulation of reactive oxygen species (ROS), and expression of senescence markers. Lysophosphatidic acid (LPA) is a growth factor‐like lipid mediator that regulates various physiological functions via activating multiple LPA G protein‐coupled receptors. Here, we study the roles of LPA and LPA receptors in premature aging. We report that the protein level of LPA_3_ was highly downregulated through internalization and the lysosomal degradation pathway in Progerin‐transfected HEK293 cells. By treating Progerin HEK293 cells with an LPA_3_ agonist (OMPT, 1‐Oleoyl‐2‐O‐methyl‐rac‐glycerophosphothionate) and performing shRNA knockdown of the *Lpa3r* transcript in these cells, we showed that LPA_3_ activation increased expression levels of antioxidant enzymes, consequently inhibiting ROS accumulation and ameliorating cell senescence. LPA_3_ was shown to be downregulated in HGPS patient fibroblasts through the lysosomal pathway, and it was shown to be crucial for ameliorating ROS accumulation and cell senescence in fibroblasts. Moreover, in a zebrafish model, LPA_3_ deficiency was sufficient to cause premature aging phenotypes in multiple organs, as well as a shorter lifespan. Taken together, these findings identify the decline of LPA_3_ as a key contributor to the premature aging phenotypes of HGPS cells and zebrafish.

## Graphical abstract

In normal cells, activation of LPA3 stabilizes Nrf2 and enhances antioxidants to prevent accumulation of reactive oxygen species (ROS) and cell senescence. In Hutchinson–Gilford progeria syndrome (HGPS) cells, LPA3 is shown to be downregulated through high internalization and subsequent lysosomal degradation. The decline of LPA3 contributes to ROS accumulation and cell senescence of HGPS cells.

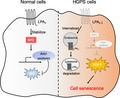

## 1 INTRODUCTION

Laminopathies are inherited degenerative disorders that are mostly related to mutations in the *LMNA* gene. This gene encodes alternative proteins, Lamin A and Lamin C, that belong to type V intermediate filaments, which are important nuclear proteins in the human body. These proteins contribute to maintaining the integrity of nuclear architecture, maintaining heterochromatin, and DNA repair (Broers, Ramaekers, Bonne, Yaou, & Hutchison, 2006). Hutchinson–Gilford progeria syndrome (HGPS) is one of the most severe laminopathies and a rare genetic disorder. It is typically caused by a silent mutation (c. 1824C > T; p. Gly608Gly) in exon 11 of *LMNA* that activates an alternative pre‐mRNA cryptic splicing donor site and causes a 150‐nucleotide deletion, which results in expression of Lamin A with 50 amino acids deleted. The missing sequence of amino acids includes the recognition site for ZMPSTE24 endoprotease, which cleaves farnesylated cysteine. Thus, the mutation leads to the accumulation of a permanently farnesylated, un‐cleaved prelamin A isoform named Progerin (Gordon, Rothman, López‐Otín, & Misteli, 2014). Patients with HGPS begin showing premature aging features resembling normal aging before 1 year of age, including wrinkled skin, atherosclerosis, and loss of eyesight. The major cause of death for these patients is cardiovascular disease, and their average lifespan is 14.6 years (Merideth et al., 2008). As a result, HGPS is studied as a model for understanding the fundamental biological processes of aging diseases. Given that increased levels of reactive oxygen species (ROS) play an important role in the developing symptoms of HGPS and normal aging (Viteri, Chung, & Stadtman, 2010), many current studies are focusing on ameliorating oxidative stress in HGPS cells (Park & Shin, 2017). Indeed, oxidative stress affects a wide range of physiological and pathological functions, and excess ROS will damage various cellular components, leading to aging‐related diseases and cancers (Cui, Kong, & Zhang, 2012).

Notably, multiple reports have demonstrated that lysophosphatidic acid (LPA) is a potent regulator of ROS (Schmitz, Thömmes, Beier, & Vetter, 2002). LPA production was found to be upregulated by oxidative stress to protect microglia cells against oxidative stress‐induced cell viability through LPA receptors (Awada et al., 2012). LPA is a bioactive lipid mediator that is mostly synthesized from lysophosphatidylcholine (LPC) by ectoenzyme lysophospholipase D (lyso‐PLD)/autotaxin (ATX). LPA exerts multiple physiological functions through six identified G protein‐coupled receptors (GPCR), LPA_1_–LPA_6_. LPA receptor knockout (KO) mice showed that LPA has various physiologically regulatory roles, as it is involved in neuronal development (Estivill‐Torrus et al., 2008), angiogenesis (Chen, Chou, Chen, & Lee, 2015), hair follicle formation (Hayashi, Inoue, Suga, Aoki, & Shimomura, 2015), and hematopoiesis (Lin et al., 2016) through different LPA receptors. LPA modulates the levels of cAMP differently in senescent fibroblasts than in young fibroblasts. This difference in response might be attributable to the change in expression levels of each LPA receptor (Jang et al., 2006). In addition, LPA signaling was shown to regulate the secretion of the inflammatory signal axis IL‐6‐STAT3 (Miyabe et al., 2014), which is also recognized as a senescence‐associated secretory phenotype (SASP) in senescent cells (Kojima, Inoue, Kunimoto, & Nakajima, 2013). Moreover, our previous studies have demonstrated that the extracellular matrix (ECM) is tightly controlled by LPA signaling (Wu et al., 2008). At the same time, ECM dysregulation, including homeostasis imbalances of collagens, proteoglycans, and MMPs, is implicated as a critical factor in disease progression of patients with HGPS (Harten et al., 2011). Together, the above evidence indicates that LPA signaling might act as an important regulator for aging phenotypes of both HGPS and normal cells.

Thus, the major goal in this study is to identify the effects of LPA and LPA receptors on the aging process of HGPS cells. To investigate the relationship between LPA and HGPS, we used a Progerin‐expressing HEK293 cell model and then HGPS patient fibroblasts in this study. LPA_3_ was shown to be downregulated consistently through the lysosomal pathway in both Progerin HEK293 cells and HGPS patient fibroblasts. Moreover, activating LPA_3_ by its agonist, OMPT, abolished ROS accumulation and rescued cell senescence of Progerin cells. Notably, *Lpa_3_^−/−^* zebrafish showed multiple premature aging phenotypes. Our findings suggested that LPA regulates ROS levels and cell senescence through LPA_3_ to alleviate cell aging in HGPS.

## 2 RESULTS

### 2.1 Overexpression of Progerin alters protein levels of LPA_2_ and LPA_3_ in HEK293 cells

**Figure 1:**
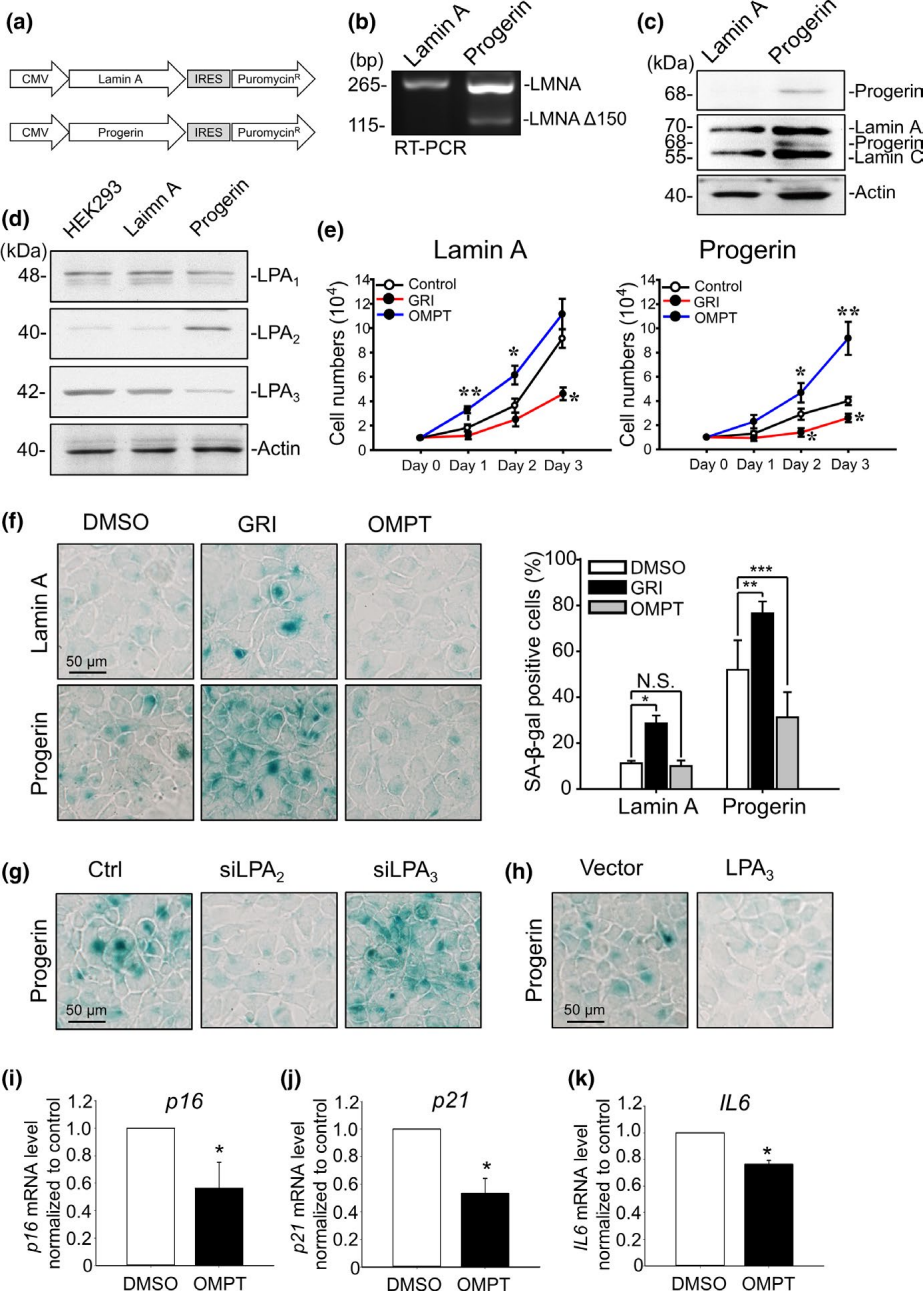
LPA_2_ upregulation and LPA_3_ downregulation correlate with attenuated cell proliferation and increased senescence in Progerin HEK293 cells. (a) Design of vectors expressing Lamin A and Progerin. Puromycin was applied as the selection marker to establish Progerin HEK293 stable cells. (b) PCR with primer targeting outside of the cryptic splicing site shows alternate 150‐nucleotide splicing of Lamin A mRNA in Progerin HEK293 cells. (c) Western blot with anti‐Lamin A/C (ab108595) and anti‐Progerin (sc‐81611) antibody shows expression of Progerin in Progerin HEK293 cells (d) Western blot with LPA receptor antibodies shows upregulation of LPA_2_ and downregulation of LPA_3_ in Progerin HEK293 cells. Actin was used as a loading control. (e) Cell number measurements show that, in both Lamin A and Progerin HEK293 cells, activating LPA_2_ with 5 μM of GRI decreased the cell proliferation rate, but activating LPA_3_ with 100 nM of OMPT increased the cell proliferation rate. (f) Representative images of senescence‐associated β‐gal staining assay and its quantified results. Treating Lamin A and Progerin HEK293 cells with 5 μM of GRI for 4 days increased the percentage of β‐gal‐positive cells in both cell lines. Treating Progerin HEK293 cells with 100 nM OMPT for 4 days reduced the percentage of β‐gal‐positive cells. (g) Knockdown of LPA_2_ by siRNA (siLPA_2_) reduced β‐gal‐positive Progerin HEK293 cells. Knockdown of LPA_3_ by siRNA (siLPA_3_) increased β‐gal‐positive cells. (h) Overexpression of LPA_3_ decreased β‐gal‐positive cells. (i) Real‐time qPCR showed that treatment of 100 nM OMPT for 24 hr reduced mRNA level of *p16* in Progerin HEK293 cells*.* (j) 100 nM OMPT for 24 hr reduced mRNA level of *p21.* (k) 100 nM OMPT for 24 hr reduced mRNA level of *IL6.* GAPDH was used as internal control. ANOVA and Student's *t* test; *p < .05, **p < .01, ***p < .001

To establish the HGPS cell model, wild‐type control *LMNA* cDNA and mutant *LMNA* cDNA carrying a single point mutation (c.1824 C > T) were stably expressed in HEK293 cells (Figure 1a). PCR with primers designed to amplify between cryptic splicing donor sites showed a 150‐nucleotide deletion of mRNA caused by a point mutation at *LMNA* c.1824 (Figure 1b). Western blot analysis with Progerin (sc‐81611) and Lamin A/C (ab108595) antibodies showed that overexpression of Progerin produced truncated Lamin A protein (Figure 1c) and led to abnormal nucleus morphology (Figure S1A). Overexpression of Progerin retarded cell proliferation and increased ROS accumulation (Figure S1B‐C). In addition, overexpression of Progerin increased the percentage of senescence‐associated β‐galactosidase‐positive (SA‐β‐gal^+^) cells (Figure S1D) and mRNA level of senescence‐related genes, including *p16*, *p21*, and *IL6* (Figure S1E‐G). These results confirmed that overexpression of Progerin indeed caused HEK293 cell senescence.

The Western blot analysis with LPA receptor antibodies showed that the protein level of LPA_2_ was significantly higher, whereas LPA_3_ was significantly lower in Progerin HEK293 cells than in parental HEK293 and Lamin A cells (Figure 1d). LPA_1_, LPA_2_, and LPA_3_ all belong to the endothelial differentiation genes (EDG) protein family and are the most studied currently (Taniguchi et al., 2017). Therefore, we set out to identify the roles of LPA_2_ and LPA_3_ in the progression of aging in Progerin cells.

### 2.2 LPA_2_ and LPA_3_ have distinct functions in regulating cell senescence in Progerin HEK293 cells

The alteration of LPA_2_ and LPA_3_ protein levels indicated that these two receptors might be involved in the cell senescence process of Progerin HEK293 cells. To investigate the functions of LPA receptors in HGPS, we applied agonists, RNA interference, and overexpression vectors of LPA receptors to Lamin A and Progerin cells in the following experiments. Two LPA receptor‐selective agonists were applied to Lamin A and Progerin HEK293 cells: LPA_2_ agonist, GRI 977143 (GRI), and LPA_3_ agonist, 2S‐OMPT (OMPT). We have used both of these common agonists in our previous research (Lin et al., 2016).

Treatment with 5 μM of GRI retarded cell proliferation in both Lamin A and Progerin HEK293 cells. In contrast, treatment with 100 nM of OMPT enhanced cell proliferation in both Lamin A and Progerin HEK293 cells (Figure 1e). Moreover, 4 days treatment with 5 μM of GRI induced higher SA‐β‐gal^+^ signals in both Lamin A and Progerin HEK293 cells, whereas 4 days treatment with 100 nM of OMPT reduced SA‐β‐gal^+^ signals in Progerin HEK293 cells (Figure 1f). We then knocked down LPA_2_ and LPA_3_ by siRNA in Progerin HEK293 cells to confirm the effects of LPAR agonists on cell senescence. Efficiency of siRNA is shown in Figure S2A. Knockdown of LPA_2_ reduced SA‐β‐gal^+^ signals, whereas knockdown of LPA_3_ increased SA‐β‐gal^+^ signals in Progerin HEK293 cells (Figure 1g). Furthermore, overexpression of LPA_3_ (Figure S2D) reduced SA‐β‐gal^+^ signals in Progerin HEK293 cells (Figure 1h). Furthermore, treatment of 100 nM OMPT to activate LPA_3_ for 1 day also reduced mRNA level of senescence‐related genes *p16*, *p21*, and *IL6* (Figure 1i–k). Immunostaining probing Lamin A/C showed that misshapen nuclei of Progerin HEK293 cells were ameliorated by 4 days treatment of OMPT. However, treatment of OMPT had no effects on recovery of Lamin B1 protein level, which is reduced by Progerin (Figure S3). Inhibition of LPA_1_ by 100 nM of LPA_1_ antagonist AM966 for 4 days had no effect on Progerin cell senescence (Figure S4A). The results above indicate that LPA_2_ and LPA_3_ oppositely regulate cell senescence in Progerin HEK293 cells.>

### 2.3 LPA_2_ and LPA_3_ have distinct functions in regulating ROS in Progerin HEK293 cells

**Figure 2:**
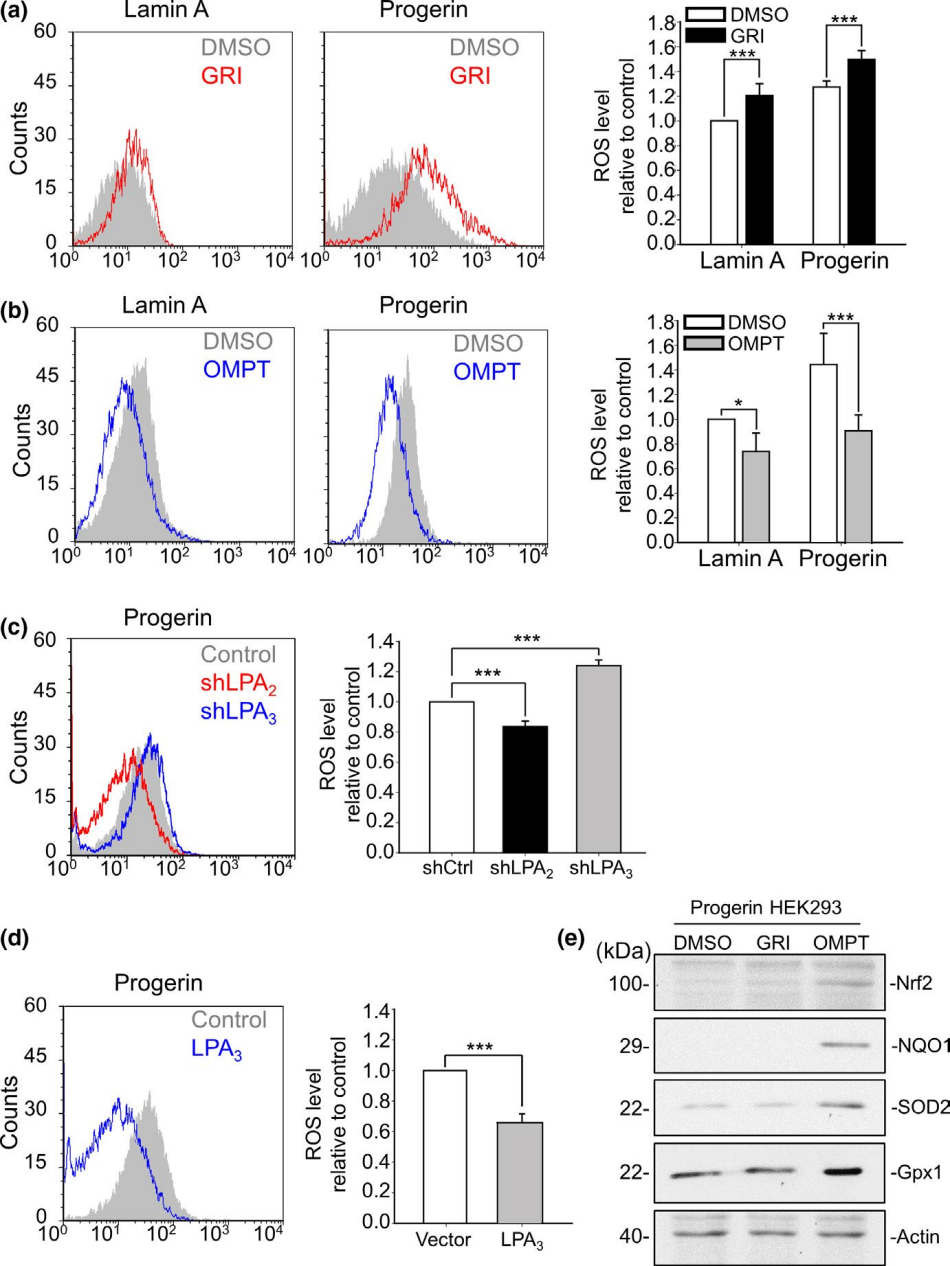
LPA_2_ increases, but LPA_3_ suppresses ROS buildup in Progerin HEK293 cells. (a) By flow cytometry, CM‐DCFDA staining results show that activating LPA_2_ by 5 μM GRI for 24 hr increased ROS levels in both Lamin A and Progerin HEK293 cells. (b) Activating LPA_3_ by 100 nM OMPT for 24 hr decreased ROS levels in both Lamin A and Progerin HEK293 cells (c) Knockdown of LPA_2_ by shRNA (shLPA_2_) reduced ROS levels, but knockdown of LPA_3_ by shRNA (shLPA_3_) increased ROS levels in Progerin HEK293 cells. (d) Overexpression of LPA_3_ reduced ROS levels in Progerin HEK293 cells. (e) After 48 hr of 5 μM GRI and 100 nM OMPT, Western blot results showed that activation of LPA_3_, but not LPA_2_, induced expression of the antioxidant mediators Nrf2, NQO1, SOD2, and Gpx1 in Progerin HEK293 cells. Actin was used as the loading control. ANOVA and Student's *t* test; *p < .05, ***p < .001

Given that oxidative stress is one of the representative characteristics of Progerin cells and senescent cells, we set out to investigate the effects of LPA receptors on ROS in Progerin HEK293 cells. Activating LPA_2_ by 5 μM of GRI for 24 hr enhanced ROS accumulation in both Lamin A and Progerin cells (Figure 2a). By contrast, activating LPA_3_ by 100 nM of OMPT reduced ROS levels in both Lamin A and Progerin cells at 24 hr (Figure 2b). Similarly, knockdown of LPA_2_ and LPA_3_ by shRNA showed opposite effects on ROS production: LPA_2_ enhanced ROS levels, whereas LPA_3_ reduced them in Progerin HEK293 cells (Figure 2c). Western blot analysis with LPAR antibodies showed reasonable knockdown efficiency of shLPA_2_ and shLPA_3_ (Sup 2B, C). In addition, overexpression of LPA_3_ reduced ROS levels in Progerin HEK293 cells (Figure 2d). To understand how these two receptors regulate ROS levels, we collected protein lysates from Progerin cells after treating them with 5 μM of GRI and 100 nM of OMPT for 48 hr. Western blot results showed that activating LPA_3_, but not LPA_2_, upregulated protein levels of Nrf2, SOD2, Gpx1, and NQO1, which are essential ROS‐eliminating enzymes, in Progerin cells (Figure 2e).

### 2.4 Decline of LPA_3_ causes ROS accumulation and cell senescence in HGPS patient fibroblasts

**Figure 3:**
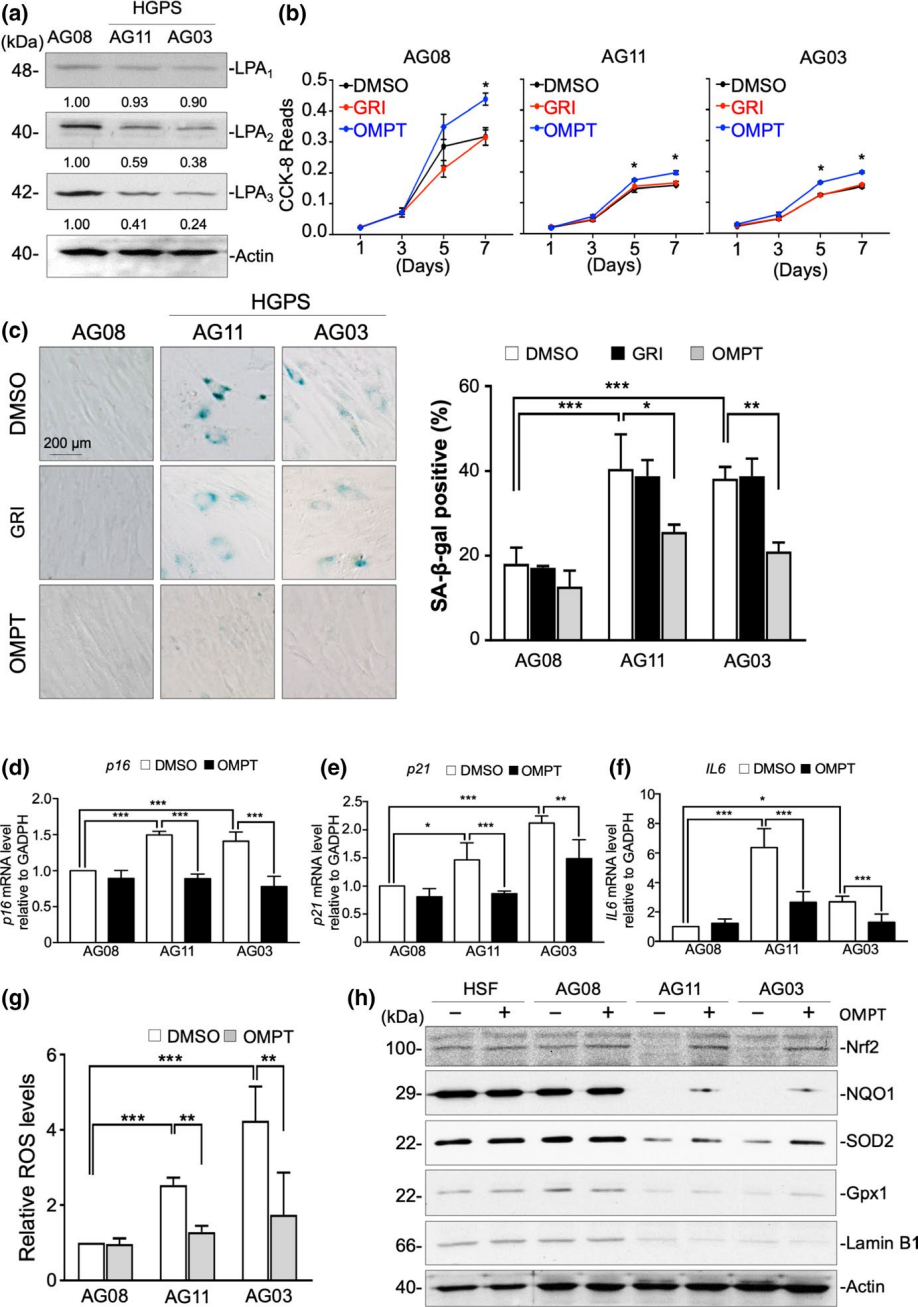
LPA_3_ reduction accelerates cell senescence in HGPS patient fibroblasts. (a) Western blot results show lower LPA_2_ and LPA_3_ protein levels in HGPS patient fibroblasts (AG11 and AG03) than in normal fibroblasts (AG08). (b) CCK‐8 assay revealed that activating LPA_3_ with 100 nM OMPT for 5 and 7 days rescued cell proliferation of HGPS AG11 and AG03 fibroblasts after. However, activating LPA_2_ with 5 μM GRI had no effect on cell proliferation. (c) Representative images of senescence‐associated β‐gal staining assay and its quantified results. Treating with 100 nM OMPT for 7 days reduced the percentage of β‐gal‐positive HGPS AG03 and AG11 fibroblasts. However, activating LPA_2_ with 5 μM GRI had no effect on cell senescence. (d) Real‐time qPCR showed that treatment of 100 nM OMPT for 24 hr reduced mRNA level of *p16* in HGPS patient fibroblasts (AG11 and AG03)*.* (e) 100 nM OMPT for 24 hr reduced mRNA level of *p21.* (f) 100 nM OMPT for 24 hr reduced mRNA level of *IL6.* GAPDH was used as internal control. (g) By flow cytometry, DCFDA staining results show that activating LPA_3_ by 100 nM OMPT for 48 hr decreased ROS levels in both HGPS AG03 and AG11 fibroblasts. However, activating LPA_2_ with 5 μM GRI had no effect on ROS level. (h) Western blot results show that activating LPA_3_ by 100 nM OMPT for 48 hr stabilized Nrf2 in HGPS AG11 fibroblasts and enhanced protein levels of NQO1, SOD2, and Gpx1, but not Lamin B1. Actin was used as a loading control. ANOVA and Student's *t* test; *p < .05, **p < .01, ***p < .001

Since the Progerin HEK293 cell model provided preliminary evidence on the roles of the LPA‐LPA receptor axis, we obtained HGPS patient fibroblasts AG11513F (AG11) and AG03199C (AG03) to compare with the previous observations. Expression of Progerin was confirmed by Western blot probing Lamin A/C and Progerin antibodies (Figure S5A). In addition to SA‐β‐gal staining, mRNA level of senescence‐related genes *p16*, *p21*, and *IL6* were measured to confirm cell senescence status of HGPS patient fibroblasts (Figure S5B). Western blot analysis showed lower levels of LPA_3_ and similar levels of LPA_1_ in both HGPS patient fibroblasts compared to age‐matched normal fibroblasts (AG08470B, AG08). LPA_2_ protein levels also decreased in HGPS patient fibroblasts, which was not consistent with Progeria HEK293 cells (Figure 3a). Moreover, a colorimetric cell viability assay Cell Counting Kit‐8 (CCK‐8) was used to determine proliferation of control and HGPS fibroblasts. Activating LPA_3_ with 100 nM of OMPT increased cell proliferation of HGPS patient and normal fibroblasts, whereas activating LPA_2_ with 5 μM of GRI had no effect on cell proliferation (Figure 3b). Immunofluorescent staining of Ki67 also showed that treatment of 100 nM OMPT for 24 hr increased Ki67‐positive cell percentages, indicating activation of LPA_3_ induces cell proliferation (Figure S6). In addition, 7 days of treatment with 100 nM of OMPT ameliorated cell senescence of HGPS patient fibroblasts, while treatment with 5 μM of GRI had no effects (Figure 3c). Furthermore, treatment of 100 nM OMPT to activate LPA_3_ in HGPS patient fibroblasts for 1 day also reduced mRNA level of senescence‐related genes *p16*, *p21*, and *IL6* (Figure 3d–f). Treatment of 2 mM NAC for 4 days in both Progerin HEK293 cells and HGPS patient fibroblasts showed amelioration of cell senescence caused by oxidative stress (Figure S7). Furthermore, treatment with 100 nM of OMPT for 2 days reduced ROS accumulation in HGPS patient fibroblasts, but 5 μM of GRI had no effects, similar to the above experiments (Figure 3g, Figure S8). As with Progerin HEK293 cells, activating LPA_3_ with 100 nM of OMPT for 2 days rescued expression levels of Nrf2, Gpx1, SOD2, and NQO1 (Figure 3h). Gpx1, SOD2, and NQO1 have been shown to be regulated at the transcriptional level by Nrf2 (Shanmugam, Narasimhan, Tamowski, Darley‐Usmar, & Rajasekaran, 2017). Nrf2 serves as a master regulator of cellular redox homeostasis by mediating the transcriptional activation of protective genes through the antioxidant response element (ARE) (Johnson et al., 2008). Notably, LPA was reported to stabilize Nrf2 to protect neuron cells in patients with Huntington's diseases from oxidative stress (Jang et al., 2019). Western blot analysis and immunofluorescent staining showed that activating LPA_3_ with OMPT rescued the protein level of Nrf2 in Progerin fibroblasts. In addition, activation of LPA_3_ also rescued nuclear translocation of Nrf2 in HGPS patient fibroblasts (Figure 3h, Figure S9). Furthermore, treatment of Nrf2 inhibitor ML385, which binds to Neh1 domain of Nrf2 to block Nrf2 transcriptional activity, was shown to cause downregulation of antioxidants and cell senescence in AG08 cells. Moreover, ML385 also abolished effect of OMPT on rescuing antioxidants and decreasing cell senescence in HGPS patient fibroblasts (Figure S10). The results provided functional link between LPA_3_ and cell senescence through Nrf2 pathway. However, treatment of OMPT had no effects on recovery of Lamin B1 protein level in HGPS patient fibroblasts (Figure 3h). These results demonstrated that the decline of LPA_3_ destabilizes the Nrf2–antioxidant pathway and increases oxidative stress in HGPS cells.

### 2.5 LPA_3_ proteins are highly internalized and sorted to the lysosome degradation pathway in Progerin HEK293 cells

**Figure 4:**
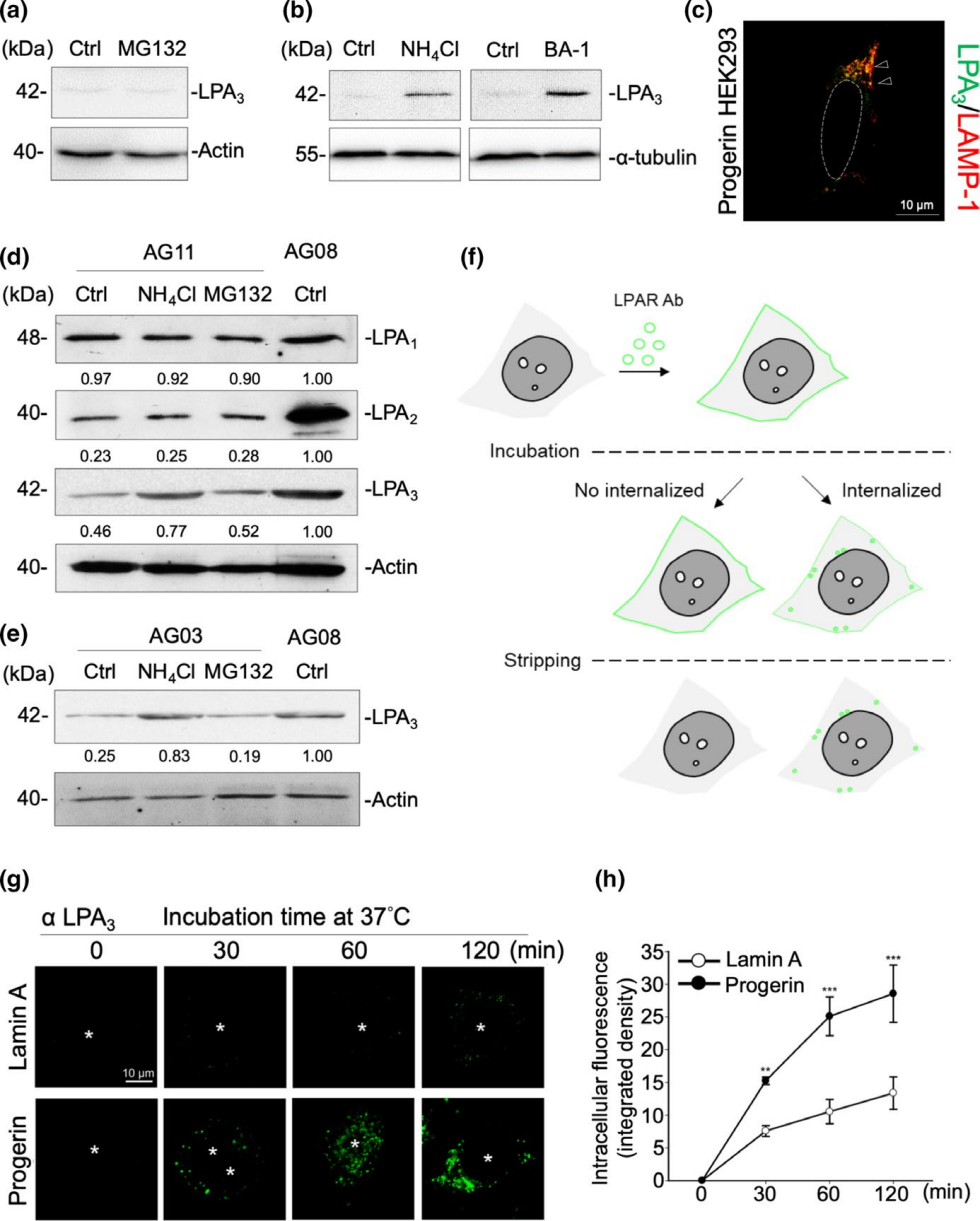
LPA_3_ is highly internalized and sorted to the lysosome degradation pathway in Progerin cells. (a) Western blot results show that treating Progerin HEK293 cells with 5 μM of MG132 for 6 hr showed no LPA_3_ protein restoration. DMSO was used as solvent control (Ctrl). (b) Western blot results show that Progerin HEK293 cells were treated with 5 mM of NH_4_Cl or 20 nM of Bafilomycin A1 (BA‐1) for 6 hr to block LPA_3_ degradation through the lysosome pathway. ddH_2_O was used as solvent control for NH_4_Cl (Ctrl in upper panel). DMSO was used as solvent control for BA‐1 (Ctrl in lower panel). (c) Western blot results show that HGPS AG11 fibroblasts were treated with 5 mM of NH_4_Cl and 5 μM of MG132 for 6 hr. NH_4_Cl treatment rescued LPA_3_, but not LPA_1_ or LPA_2_, in HGPS AG11 fibroblasts. ddH_2_O and DMSO were both loaded to control sample as solvent control (Ctrl). (d) Counter‐immunofluorescent staining by LPA_3_ and LAMP‐1 shows that LPA_3_ localized into the lysosomes in Progerin HEK293 cells. (e) Western blot results show that NH_4_Cl treatment rescued LPA_3_ in HGPS AG03 fibroblasts. ddH_2_O and DMSO were both loaded to control as solvent control (Ctrl). (f) Scheme representing the internalization assay of LPA receptors_._ (g) Representative images of time‐dependent LPA_3_ internalization. Internalization of LPA_3_ was higher in Progerin HEK293 cells. (h) Integrated density of intracellular fluorescence was quantified to indicate internalized LPA_3_. ANOVA and Student's *t* test; **p < .01, ***p < .001

Although we observed downregulation of LPA_3_ protein levels in Progerin HEK293 cells, real‐time qPCR analysis showed increased levels of *Lpar3* mRNA in Progerin cells than in Lamin A cells (Figure 1d, Figure S11A). We speculated that the downregulation of LPA_3_ occurs at the post‐translational level. First, measuring the mRNA stability of *Lpar3* showed no differences between Lamin A and Progerin cells (Figure S11B). Notably, having demonstrated the constitutive internalization of GPCR over time, Alcántara‐Hernández et al. showed that these receptors will reach the lysosomal compartment for degradation (Alcántara‐Hernández, Hernández‐Méndez, Campos‐Martínez, Meizoso‐Huesca, & García‐Sáinz, 2015). Treatment with 20 mM of NH_4_Cl and 20 nM of Bafilomycin A1, but not with 5 μM of MG132, rescued LPA_3_ degradation in Progerin HEK293 cells (Figure 4a–b). Furthermore, consistent with previous results, the lysosome inhibitor NH_4_Cl, but not the proteasome inhibitor MG132, rescued protein levels of LPA_3_, but not LPA_1_ or LPA_2_, in HGPS patient fibroblasts AG11 (Figure 4c). Likewise, the lysosome inhibitor NH_4_Cl, but not the proteasome inhibitor MG132, rescued protein levels of LPA_3_ in HGPS patient fibroblasts AG03 (Figure 4e). In Progerin cells, we found that internalized LPA_3_ entered the lysosomes, which we confirmed by overlapping the immunofluorescent signals of LPA_3_ and lysosomal marker LAMP‐1 (Figure 4d). Next, we determined the internalization of LPA receptors of both Lamin A and Progerin HEK293 cells by an LPA receptors internalization assay (Figure 4f). We found that the internalization of LPA_3_, but not LPA_2_, increased in Progerin cells (Figure 4g–h, Figure S13). This indicates that the difference in LPA_3_ protein levels between Lamin A and Progerin cells is caused by the greater internalization of LPA_3_ in Progerin cells. Together, this evidence indicates that the decline of LPA_3_ in Progerin cells is due to enhanced internalization and lysosomal degradation.

### 2.6 *Lpa_3_^−/−^* zebrafish shows premature aging phenotypes

**Figure 5:**
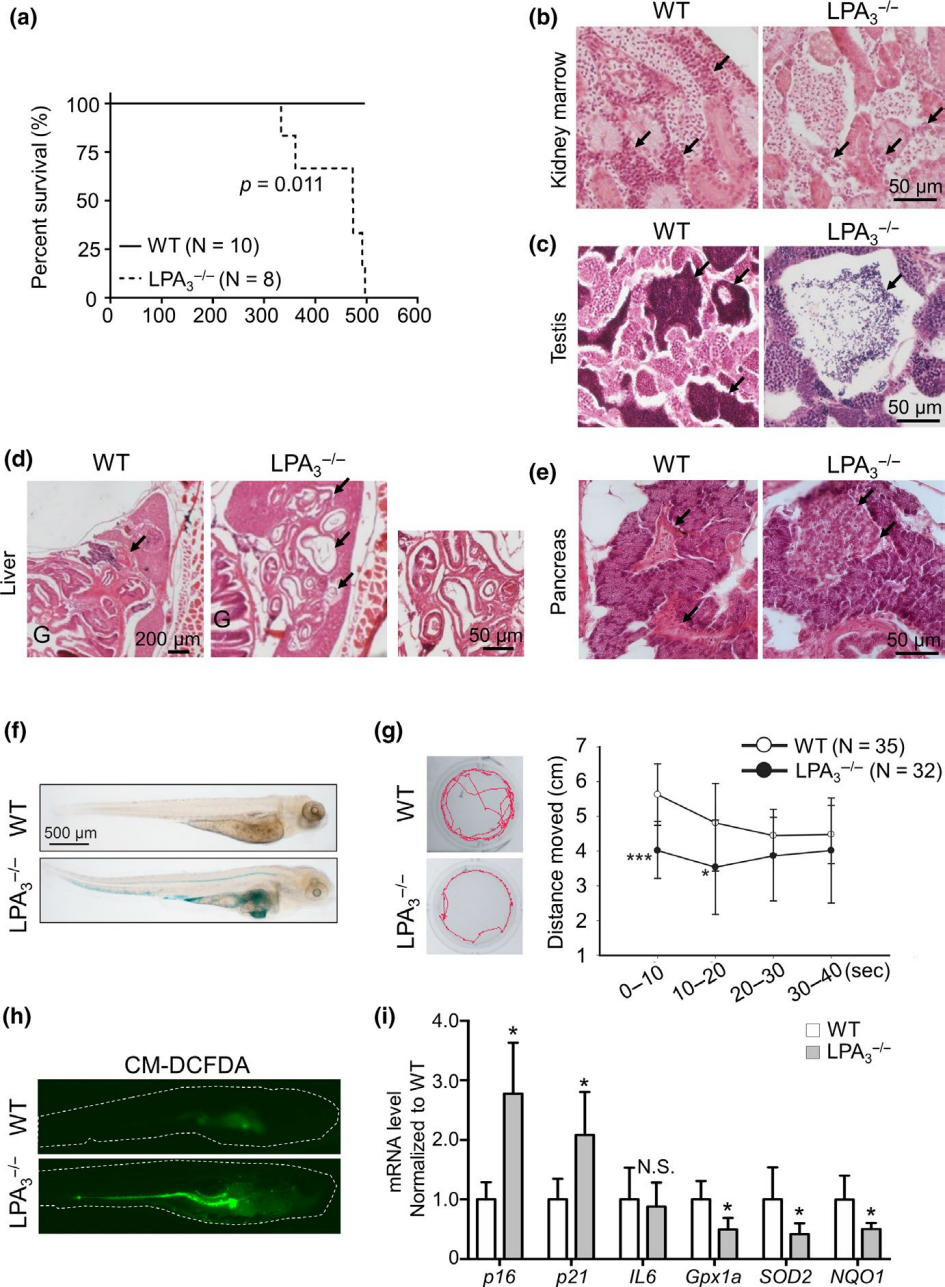
LPA_3_ deficiency in zebrafish leads to premature aging phenotypes. (a) Kaplan–Meier survival analysis demonstrated a shorter lifespan in *Lpa_3_^−/−^* zebrafish than in corresponding WT siblings (*p* value = .011, *N* > 8). (b) Histological analysis shows hematopoietic stem cells loss in kidney marrow of 6‐month‐old *Lpa_3_^−/−^* zebrafish. (c) Sperm loss in the testis. (d) Bile duct hyperplasia. (e) Islet hyperplasia in the pancreas. (f) By senescence‐associated β‐gal staining assay, *Lpa_3_^−/−^* larvae show a positive β‐gal signal. (g) Representative images of zebrafish swimming tracks obtained and analyzed by locomotor. Locomotor results show that *Lpa_3_^−/−^* larvae had lower mobility than WT upon drastic light onset and offset. (h) CM‐DCFDA staining showed increased ROS levels in 5 dpf *Lpa_3_^−/−^* larvae. (i) Real‐time qPCR showed enhanced *p16* and p21, whereas reduced *Gpx1a*, *SOD2*, and *NQO1* of 5 dpf *Lpa_3_^−/−^* larvae. β‐Actin was used as internal control. ANOVA and Student's *t* test; *p < .05, ***p < .001

To further investigate the roles of *Lpa_3_* in the aging progress, we took advantage of an *Lpa_3_^−/−^* zebrafish model established in our previous research (Lin et al., 2018). Kaplan–Meier survival analysis revealed that *Lpa_3_^−/−^* zebrafish have shorter lifespans than wild‐type zebrafish (Figure 5a). Accumulated oxidative stress in aging individual will lead to multiple organs impairment. For instance, high oxidative stress reduces hematopoietic stem cells (HSC) of aged individuals and disrupts homeostasis of blood components (Richardson, Yan, & Vestal, 2015). To elucidate the effects of LPA_3_–antioxidants axis signaling impairment on different organs, we analyzed 6‐month‐old adult male zebrafish histologically with H&E staining. The number of HSC, which locates in kidney marrows of zebrafish, was lower in *Lpa_3_^−/−^* zebrafish than in corresponding wild‐type zebrafish (Figure 5b). This indicates that disrupting LPA_3_ signaling impairs the supply of blood cells, which agrees with our previous study (Chiang et al., 2011). Moreover, similar to HSC, sperm is also susceptible to oxidative stress (Sabeti, Pourmasumi, Rahiminia, Akyash, & Talebi, 2016). The testis of *Lpa_3_^−/−^* zebrafish showed reduced sperm production (Figure 5c), consistent to age‐dependent loss of sperm in LPA_3_ knockout male mice (Ye, Skinner, Kennedy, & Chun, 2008). Besides, bile duct hyperplasia was reported as liver damage phenotype when animal exposed to oxidative stress (Eldridge et al., 2014). Interestingly, bile duct hyperplasia was also observed in *Lpa_3_^−/−^* zebrafish (Figure 5d). Furthermore, pancreatic *β* cells are vulnerable to oxidative stress due to its high endogenous ROS level and low expression of antioxidant enzymes (Wang & Wang, 2017). Therefore, high accumulation of oxidative stress will lead to abnormally pancreatic islets hyperplasia, which is also observed in the *Lpa_3_^−/−^* zebrafish (Figure 5e). Moreover, five dpf (days postfertilization) *Lpa_3_^−/−^* zebrafish larvae showed a higher intensity of SA‐β‐gal staining than wild‐type (Figure 5f). Moreover, 5 dpf *Lpa_3_^−/−^* zebrafish larvae showed lower mobility after vibration induction than WT (Figure 5g). Furthermore, CM‐DCFDA staining results revealed that ROS level in 5 dpf *Lpa_3_^−/−^* zebrafish larvae is higher than WT (Figure 5h). Moreover, mRNA level of antioxidant enzyme genes, including *Gpx1a*, *SOD2*, and *NQO1*, was significantly lower in 5 dpf *Lpa_3_^−/−^* zebrafish larvae. In addition, mRNA level of *p16* and *p21* was also higher in 5 dpf *Lpa_3_^−/−^* zebrafish larvae (Figure 5i). These data from *Lpa_3_^−/−^* zebrafish models provide further in vivo evidence for the role of LPA_3_ decline as a driving signal for misregulation of oxidative stress aging processes globally.

### 2.7 Knockdown of LPA_3_ sensitizes normal fibroblasts to oxidative stress

**Figure 6:**
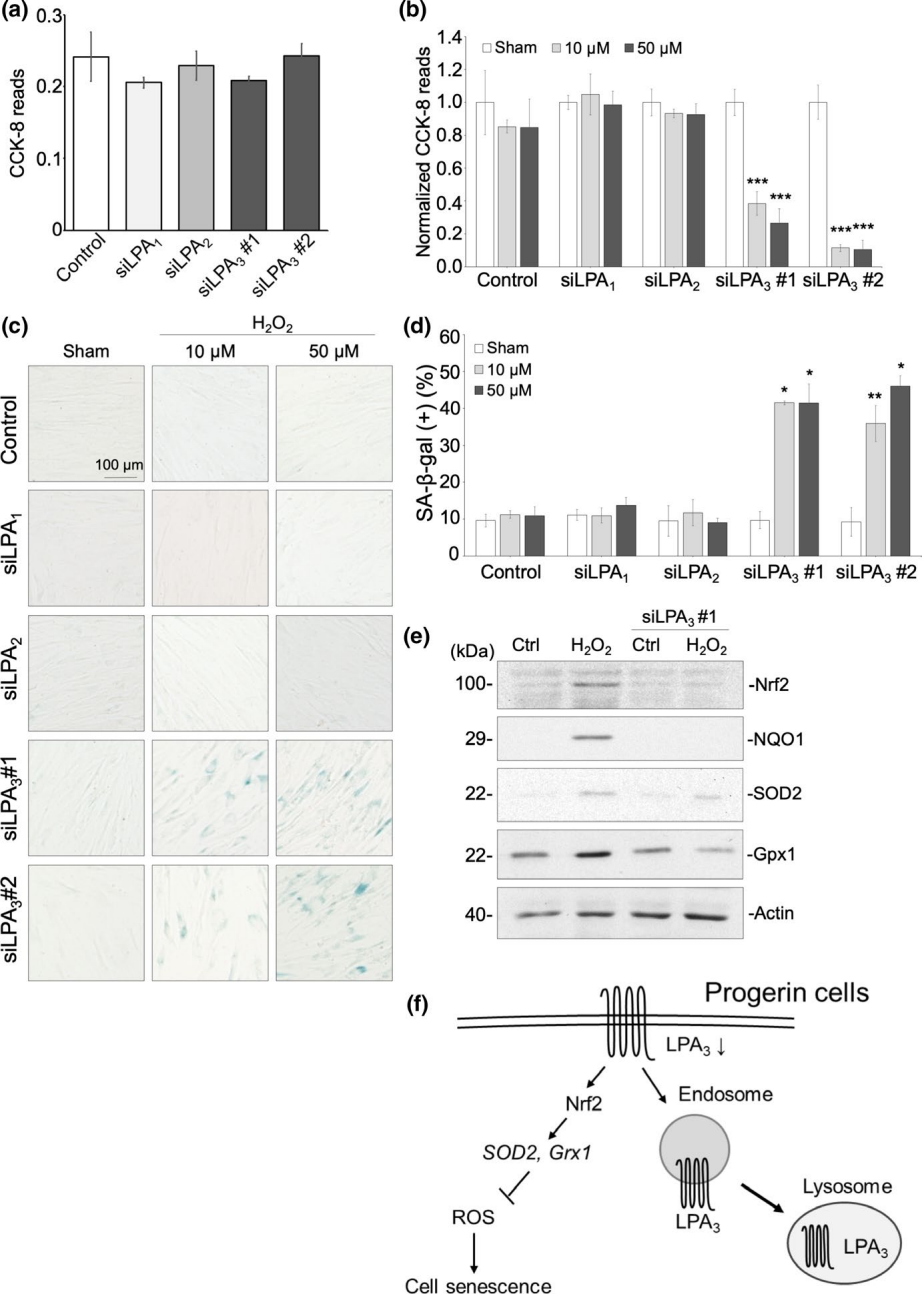
LPA_3_ protects fibroblasts against cell senescence caused by oxidative stress. (a) CCK‐8 assay showed that knockdown of LPA receptors alone had no effect on cell proliferation. (b) CCK‐8 revealed that treatment with 10 μM and 50 μM H_2_O_2_ for 3 days significantly decreased cell proliferation in knockdown of LPA_3_, but not of LPA_1_ or LPA_2_. CCK‐8 reads were normalized to a sham group. (c) Representative images of senescence‐associated β‐gal staining assay. Treatment with 10 μM and 50 μM H_2_O_2_ for 3 days significantly increased percentages of β‐gal + cells in knockdown of LPA_3_, but not of LPA_1_ or LPA_2_. Quantification was shown in (d). (e) Western blot showed that 100 μM of H_2_O_2_ treatment for 6 hr increased protein level of Nrf2, NQO1, SOD2, and Gpx1, whereas LPA_3_ knockdown abolished the effects. (f) Graphical abstract of this study. ANOVA and Student's *t* test; *p < .05, **p < .01, ***p < .001

Since *Lpa_3_^−/−^* zebrafish showed premature aging phenotypes, we performed siRNA knockdown of LPA_3_ in normal human skin fibroblasts (HSF) to emphasize the correlation between aging progress and LPA_3_ decline. Each LPA receptor was knocked down by siRNA and shown by Western blot (Figure S2a). SA‐β‐gal staining showed that normal HSF is resistant to 10 and 50 μM of H_2_O_2_, whereas not to 100, 200, and 400 μM of H_2_O_2_ (Figure S14). Cell proliferation was measured by a colorimetric cell viability CCK‐8 assay. Although knockdown of LPA_1‐3_ alone had no effect on cell proliferation (Figure 6a), LPA_3_ knockdown HSF showed decreased cell proliferation when exposed to 10 and 50 μM of H_2_O_2_ for 3 days that were tolerable for control, LPA_1_ and LPA_2_ knockdown HSF (Figure 6b). In addition, LPA_3_ knockdown fibroblasts showed higher percentages of β‐gal^+^ cells under 10 and 50 μM H_2_O_2_ treatment (Figure 6c, d). In normal HSF, Western blot showed that Nrf2 and antioxidant enzyme gene axis were increased to response to 100 μM of H_2_O_2_ treatment for 6 hr. However, LPA_3_ knockdown HSF failed to upregulate Nrf2 and antioxidant enzyme gene axis with 100 μM of H_2_O_2_ treatment (Figure 6e). Taken together, the results showed that attenuation of LPA_3_ activity will compromise cellular resistance to oxidative stress and accelerate cell senescence. This is consistent with observations from HGPS cells that LPA_3_, but not LPA_1_ or LPA_2_, is essential to resisting cell senescence caused by oxidative stress.

## 3 DISCUSSION

In this study, we found that LPA_3_ diminished ROS levels by upregulating antioxidant enzymes and consequently rescued cell senescence of Progerin cells (Figure 6f). First, we established Progerin HEK293 cells, showing that the protein level of LPA_2_ increased, while LPA_3_ decreased. Furthermore, by cell proliferation and senescence‐associated β‐galactosidase staining, we showed that LPA_2_ and LPA_3_ oppositely regulated cell senescence of Progerin HEK293 cells. DCFDA staining showed that activating LPA_2_ increased ROS levels, but activating LPA_3_ decreased ROS levels in Progerin HEK293 cells by upregulating the ROS scavenger genes *Nrf2*, *NQO1*, *SOD2,* and *Gpx1*. We hypothesized that LPA_2_ increases ROS levels to accelerate the aging progress of Progerin HEK293 cells, while LPA_3_ ameliorates it. Accordingly, when we tested our preliminary results in HGPS patient fibroblasts, only LPA_3_ activation rescued senescent phenotypes. LPA_2_ was not upregulated in HGPS patient fibroblasts. Also, LPA_2_ had no effect on ROS accumulation and cell senescence in HGPS patient fibroblasts. Similarly, *Lpa_3_^−/−^* zebrafish showed premature aging phenotypes, which emphasize the role of LPA_3_ in protecting against the cell aging process. Next, knockdown of LPA_3_ by siRNA in normal skin fibroblasts showed that LPA_3_ is indeed essential for preventing cell aging by providing resistance against oxidative stress. Notably, the decline of LPA_3_ in Progerin HEK293cells was demonstrated to be highly internalized and sorted to the lysosomal degradation pathway. To sum up, we identified the reduction of LPA_3_ signaling as a driver for oxidative stress and aging in both HGPS cell and zebrafish models. Also, LPA_3_ agonists were shown to improve effects against oxidative stress and cell senescence in HGPS cell models.

Some previous studies have suggested the correlation between aging phenotypes and LPA signaling. For instance, significantly higher concentrations of LPA were detected in the cerebral cortex synaptosomes of aged rats (Pasquare, Gaveglio, & Giusto, 2009). Also, the LPA‐LPA1 axis was determined to regulate depression in elderly populations (Moreno‐Fernandez et al., 2018). Moreover, suppressing LPA_2_ in a mouse neuron cell line alleviated heat‐induced cell apoptosis (Kortlever, Brummelkamp, Meeteren, Moolenaar, & Bernards, 2008). Furthermore, LPA was shown to modulate cAMP levels in senescent fibroblasts differently than in young fibroblasts (Jang et al., 2006). In line with our observations, knockdown of LPA_3_ led to cell senescence in mesenchymal stromal cells (MSC) (Kanehira et al., 2017). Taken together, these studies and our results support the idea that LPA signaling is a potent signaling pathway with wide‐ranging effects on the aging process.

Notably, LPA‐LPAR signaling is also influential in tumor progression. Our previous studies illustrated that LPA reduces autophagy caused by nutrient depletion (Chang, Liao, Huang, & Lee, 2007) and activates LPA_3_ to increase VEGF‐A and VEGF‐C for tumoral lymphangiogenesis in prostate cancer. In addition, multiple groups have shown that LPA mediates epithelial–mesenchymal transition to stimulate breast cancer cell invasion and metastasis (Cho, Jeong, Park, & Lee, 2019). Moreover, LPA_3_ signaling has been demonstrated to be essential for the proliferation of various cell types (Zuo et al., 2018). Cellular replication, which is limited by cell senescence, is tightly regulated by multiple tumor suppressors and oncogenes. Bypassing cell senescence appears to be crucial for the growth and expansion of cancers (Li & Chen, 2018). Accordingly, we speculate that consistently reducing LPA_3_ in various senescent cell lines might prevent tumor development.

A likely scenario, based on this study and others, for how different LPA receptors contribute to LPA signaling suggests that cells might alter the expression levels of different LPA receptors under distinct cell statuses, thereby activating various downstream pathways using same ligand, LPA. For instance, the expression level of LPA_2_ is upregulated, but LPA_3_ is downregulated, during megakaryocytes lineage differentiation from chronic leukemia cell model K562 cells by phorbol 12‐myristate 13‐acetate induction (Ho et al., 2015). Furthermore, LPA modulates the levels of cAMP differently in senescent fibroblasts from young fibroblasts due to the change of expression levels of each LPA receptor (Jang et al., 2006). Together, we conclude that, in addition to altering LPA concentration, LPA receptors’ expression levels are regulated to activate the signaling pathway to let cells exert distinct physiological functions, such as cell differentiation and the prevention of cell senescence.

We showed that the protein level of LPA_3_ is decreased in both Progerin HEK293 cells and HGPS patient fibroblasts due to high internalization of LPA_3_ in Progerin cells. Similarly, senescent human umbilical vein endothelial cells (HUVEC) (passages 10) also showed a reduction in LPA_3_, but not LPA_1_ nor LPA_2_ (Figure S15). Although LPA_1_ and LPA_2_ both belong to the EDG protein family like LPA_3_, the protein levels of LPA_1_ and LPA_2_ are not consistently observed to be downregulated by lysosomal pathways in different senescent cells. A previous study showed that LPA_3_ is mostly phosphorylated and internalized after induction, but LPA_1_ and LPA_2_ require a longer time and a larger induction to induce internalization (Alcántara‐Hernández et al., 2015). It has been suggested that PDZ domains on the C‐terminal of LPA_1_ and LPA_2_ might be responsible for prolonged cell surface resident time and reduced rates of internalization (Marchese, Paing, Temple, & Trejo, 2008). PDZ domains are protein–protein recognition modules that bind to C‐terminal short and linear PDZ ligand sequences. Notably, δ‐opioid receptor chimera with a non‐native PDZ showed delayed internalization and prolonged protein half‐life (Puthenveedu & von Zastrow, 2006). However, a tyrosine‐based motif in GPCR’s C‐terminal is considered to regulate the internalization of GPCR. Therefore, a tyrosine‐based motif recognized in LPA_3_’s C‐terminal might be important for regulating internalization and recycling in HGPS cells (Marchese et al., 2008).

Current studies mainly aim to clear Progerin to ameliorate abnormalities in nuclear shape. Farnesyltransferase inhibitors (FTI) have been reported to reverse nuclear blebbing both in vitro and in vivo (Yang, Qiao, Fong, & Young, 2008). Treatment with FTIs also improved body weight and survival rates of *Zmpste24^−^* ^/^ *^−^* (Fong et al., 2006) and HGPS‐targeted mutant mice (Yang et al., 2006). However, FTI‐treated mice still showed abnormal body weight curves and low amelioration of rib features. It is possible that alternative prenylation of prelamin A by geranylgeranyltransferase I after FTI treatment may lead to further limited benefits from FTIs (Whyte et al., 1997). Since beneficial effects of LPA_3_ on cell aging are consistent across different cell lines, identifying LPA_3_ agonists may be a high‐value health‐promoting drug discovery in the future. However, such lipid‐like ligands have problems in stability and solubility upon treatment. Most of the current available LPA‐based drug candidates are limited to lipid‐like ligands, which is understandable due to the hydrophobic environment of the LPA G protein‐coupled receptor ligand binding pockets (Kiss et al., 2012). Therefore, it is beneficial to develop specific nonlipid LPA_3_ agonists for potential therapeutics.

## 4 EXPERIMENTAL PROCEDURES

### 4.1 Cell culture and pharmacological reagents

An HEK 293 Homo sapiens embryonic kidney cell line obtained from ATCC (Manassas, VA, USA) was maintained in Dulbecco's Modified Eagle's Medium (DMEM) supplemented with 10% fetal bovine serum (FBS) (Thermo Fisher Scientific Hyclone, Waltham, MA, USA) and 1% penicillin/streptomycin. HGPS fibroblast lines AG11513F (passage 5) and AG03199C (passage 5), and age‐matched control fibroblast line AG08470B (passage 4) were received from the Coriell Institute (Camden, NJ, USA) and were maintained in minimal essential medium—Eagle's balance salts supplemented with 15% FBS. Normal human skin fibroblasts (HSF, passage 9) derived from neonatal foreskin were maintained in the same culture condition. All the experiments for progeria fibroblasts and HSF were performed before passage 12. Human umbilical vein endothelial cells (HUVEC) were obtained and prepared as described before (Hsia et al., 2017). Briefly, HUVEC harvested from human umbilical veins were plated on a culture dish and maintained in endothelial growth medium (EGM‐2 BulletKit, Lonza, Basel, Switzerland). HEK 293 and HUVEC cells were cultured at 37°C in a humidified atmosphere of 5% CO_2_. Normal human skin fibroblasts, HGPS patient fibroblasts AG11513F and AG03199C, and age‐matched control fibroblasts AG08470B were cultured under 3% oxygen in H35 HEPA Hypoxystation (Don Whitley Scientific, UK). 1‐Oleoyl‐2‐O‐methyl‐rac‐glycerophosphothionate (OMPT, Cayman Chemicals, Ann Arbor, MI, USA), GRI compound 977143 (GRI, Millipore Sigma, Burlington, MA, USA), AM966 (Cayman Chemicals), Bafilomycin A1 (Millipore Sigma), MG‐132 (Millipore Sigma), Actinomycin D (Millipore Sigma), and ML385 (Millipore Sigma) were dissolved in DMSO. Diphenyleneiodonium chloride (DPI, Millipore Sigma), N‐acetyl cysteine (NAC, Millipore Sigma), and ammonium chloride (NH_4_Cl, Millipore Sigma) were dissolved in sterile water.

### 4.2 Plasmid construct, RNA interference, and transfection

First, the PCR product of the LMNA gene was cloned into a pAS_2_V_1_ vector containing a puromycin‐resistant gene and amplified in E.coli (Strain: TOP10), followed by mini‐preparation (Geneaid, Taipei, Taiwan). Then, the complementary primers were used in the second PCR reaction to obtain a point mutation of the LMNA gene, Progerin. Sequences were confirmed by double‐strained sequencing (PURIGO Biotechnology, Taipei, Taiwan). Lamin A/Progerin overexpression vectors were then transfected into HEK 293 cells with Lipofectamine 2000 (Thermo Fisher Scientific) and selected by puromycin at 2 μg/ml. LPA_2_ shRNA (TRCN0000221131), LPA_3_ shRNA (TRCN0000356890), and a backbone control vector (pLKO.1‐puro) were purchased from the RNAi Core of Academia Sinica (Taipei, Taiwan) and transfected to HEK 293 cells by Lipofectamine 2000. LPA_1_, LPA_2_, and LPA_3_ siRNA were synthesized by Invitrogen (Thermo Fisher Scientific) and transfected to AG08470B cells by Lipofectamine 2000. Primer and siRNA sequences are listed in Figure S18.

### 4.3 Western blot

Cells were washed with ice‐cold phosphate‐buffered saline (PBS) and scratched on ice using a lysis buffer (10 mM HEPES, 1.5 mM MgCl2, 10 mM KCl, 0.5 mM DTT, 0.05% NP40, pH 7.9) with a protease inhibitor cocktail (Millipore Sigma). Lysates were incubated on ice for 15 min and then centrifuged at 4°C and 14,000 rpm for 15 min. Supernatants were collected for cytometry protein, and then, the pallets were collected for nucleus protein. For total lysates, cells were first washed with ice‐cold PBS, followed by re‐suspended in 2x Laemmli sample buffer (Bio‐Rad) containing 5% 2‐Mercaptoethanol (Millipore Sigma) with a protease inhibitor cocktail (Millipore Sigma), followed by sonication with the Digital Sonifier Cell Disruptor (Emerson, Saint Louis, MO, USA). Cycling conditions for sonication were 15 cycles of 20 s on/30 s off. Equal concentrations of samples defined by a Bradford protein assay (Bio‐Rad) were separated by 10% SDS‐polyacrylamide gel electrophoresis (PAGE) and then transferred to polyvinylidene fluoride membranes (Millipore Sigma). The membranes were blocked in TBST containing 5% nonfat dry milk, followed by incubation with specific primary antibodies diluted with TBST containing 5% bovine serum albumin (BSA, Millipore Sigma) at 4°C overnight. The membranes were then washed three times with TBST and incubated with a horseradish peroxidase‐conjugated secondary antibody at room temperature for 1 hr. Then, membranes were mixed with ECL reagents (Thermo Fisher Scientific) and imaged by X‐ray films or UVP ChemStudio Plus (Analytikjena, Jena, Germany). The band intensities were analyzed by ImageJ. Raw data for Western blot images are shown in Figures S16 and S17. Antibodies used in this study are listed in Figure S19.

### 4.4 Flow cytometry

Intracellular reactive oxygen species (ROS) were detected by flow cytometry using 2’, 7’‐dichlorofluorescein diacetate (CM‐H_2_DCFDA, Millipore Sigma), a cell‐permeable dye that emits green fluorescence after redox reaction with ROS. Cells were first cultured in 6‐well plates overnight, followed by treatment with 5 μM GRI or 100 nM OMPT. Then, cells were trypsinized and re‐suspended in PBS. Cells were stained by 10 μM CM‐H_2_DCFDA for 15 min in a 37°C cell incubator and then washed in PBS. The green fluorescence signal was then detected by the green fluorescent channel using Cyflow flow cytometry (Partec, Nürnberg, Germany). For each experiment, 10 000 cells were measured and quantified.

### 4.5 Cell proliferation

For HEK293 cell proliferation, cells were seeded in 24‐well culture plates with 1.0 × 10^4^ cells per well. After culturing overnight, the medium was replaced with medium containing LPA agonists, 5 μM GRI, or 100 nM OMPT. Cells were counted with a hemocytometer at indicated times. For HGPS patient fibroblasts, cell proliferation was evaluated by the Cell Counting Kit‐8 (CCK‐8, Dojindo Molecular Technologies, Rockville, MD, USA) according to manufacturers’ instructions. Cells were first seeded in a 96‐well plate at 2.5 × 10^3^ cells per well overnight and then treated with LPA receptor agonists. After treatment with GRI or OMPT for 1–7 days, a solution of 10 μl of CCK‐8 with 90 μl of culture medium was added at the indicated time and incubated for 1 hr to develop the CCK‐8 signal. Then, the absorbance at 450 nm wavelength was determined by a Spark^®^ multimode microplate reader (TECAN, Männedorf, Switzerland).

### 4.6 Senescence‐associated beta‐galactosidase assay

Cells were fixed with 2% formaldehyde and 0.2% glutaraldehyde in PBS. Then, the staining procedure was performed in the staining solution of X‐gal (pH = 5.9–6.1) at 37°C for at least 24 hr. After blue color was fully developed, percentage of blue SA‐β‐gal‐positive cells were counted under a microscope. To prepare the staining dye, 0.1% of X‐gal (stock: 1% in DMSO, Millipore Sigma), 5 mM potassium ferrocyanide (stock: 0.5 M, Millipore Sigma), 5 mM potassium ferricyanide (stock: 0.5 M, Millipore Sigma), 150 mM sodium chloride (NaCl, Millipore Sigma), and 2 mM magnesium chloride (MgCl_2_, Millipore Sigma) were added to 40 mM citric acid/sodium phosphate solution (stock: 400 mM, Millipore Sigma). The pH value was verified as 5.9–6.1 and adjusted by 0.1 M citric acid, if necessary. The SA‐β‐gal‐positive cells stained blue‐green color and were scored under a Zeiss AxioImager bright‐field microscopy. Results were generated from at least three independent experiments. More than 500 cells were counted in each experimental condition.

### 4.7 Internalization and immunofluorescent staining

For the LPA_2_ and LPA_3_ internalization assays, Lamin A and Progerin HEK293 cells were first cultured on glass slides overnight. Then, LPA receptors on the cell surface were labeled by antibodies that recognize the N‐terminal of LPA receptor proteins (outside the plasma membrane) and prepared in serum‐free DMEM containing 1% BSA, for 1 hr on ice. After washing three times by serum‐free DMEM, glass slides were returned to a 37°C incubator. Glass slides were then collected at serial time points for surface antibody retrieval using an acidic medium (0.5% acetic acid, 0.5M NaCl, 0.05% BSA, pH = 4.0), followed by 2% paraformaldehyde (PFA, Millipore Sigma) fixation for 10 min and permeabilized by 0.5% Triton X (Millipore Sigma) for 5 min. Internalization assay of LPA_3_ without surface antibody retrieval was shown in Figure S12. The internalized antibody was then labeled using a secondary antibody, anti‐rabbit IgG conjugated with Alexa 488. For immunofluorescent staining, Progerin HEK293 cells were cultured on glass slides overnight, then fixed by 2% PFA, and permeabilized by 0.5% Triton X for 5 min. After PBS wash, glass slides were incubated with primary antibodies for 2 hr and then labeled with fluorescent secondary antibodies for 1 hr. Fluorescent images were developed by Zeiss LSM 880 confocal microscopy. Antibodies used in this study are listed in Figure S19.

### 4.8 Establishment and maintenance of LPA_3_ knockout Zebrafish

Establishment of LPA_3_ knockout zebrafish was described in Figures S1–S19. For genotyping, we extracted genomic DNA (gDNA) from the tail fins of 1‐month‐old zebrafish using a digesting buffer (1 mg/ml proteinase K in TE buffer). Genotyping was performed by Sanger sequencing (PURIGO Biotechnology). Primers to amplify gDNA for Sanger sequencing are as follows: F’ataactgaaaccctttagacccac; R’ccaatttagagcacagatccagac. Zebrafish used in the present study were maintained following the zebrafish caring standard in the National Taiwan University Zebrafish Core Laboratory (Taipei, Taiwan).

### 4.9 Histological analysis of adult zebrafish

Six‐month‐old adult zebrafish were sacrificed by cold 4 mg/ml of *N*‐(Tris[hydroxymethyl]methyl) glycine (Tricaine, Merck, Kenilworth, NJ, USA) and then immediately fixed for 1 week with Bouin solution (75% saturated picric acid, 10% formaldehyde, 5% glacial acetic acid). After fixation, zebrafish were transferred to 70% ethanol to remove excess picric acid. For paraffin embedding, zebrafish were gradually dehydrated as follows: 70% ethanol for 1 hr, 80% ethanol for 1 hr, 90% ethanol for 1 hr, and twice absolute ethanol for 1 hr. Then, zebrafish were incubated at 60°C and infiltrated as follows: first, cleared by xylene for 15 min; second, cleared by 50% xylene and 50% soft paraffin for 15 min; and finally, infiltrated by hard paraffin for 25–30 min. Lastly, zebrafish were immediately embedded in hard paraffin and then properly trimmed to cutting size. Paraffin blocks were then sectioned at 7 μm by microtome. Histological hematoxylin–eosin (H&E) staining of the sections was subsequently performed using standard protocols. Sections were mounted with Fisher Chemical^TM^ Permount^TM^ mounting medium (Thermo Fisher Scientific) and observed using an Olympus IX73 inverted microscope system.

### 4.10 Zebrafish larvae mobility

Zebrafish larvae were kept in groups at 26–28°C in a light:dark rhythm of 14:10 hr. Each treatment group of 4 days postfertilization (dpf) zebrafish larvae was transferred into an individual well of 24‐well plates before the experiments. To detect alterations in the mobility of zebrafish larvae between different genotypes, we used the DanioVision^TM^ observation system (Noldus, Wageningen, Netherlands) to monitor and analyze the swimming tracks of zebrafish larvae. A discrete tap at the bottom of the plate holder creates a vibration in the water that evokes a startle response from the zebrafish larvae. The zebrafish larvae's tracks were recorded continually for 1 min after tap stimulation, and the swimming distances were summed every 10 s for quantification.

### 4.11 Reverse transcription (RT) and real‐time qPCR

RNA was extracted from cells using TRIZOL (Thermo Fisher Scientific Invitrogen). Complementary DNA was synthesized with 1 µg total RNA using Toyobo RT‐PCR kit. (Toyobo, Osaka, Japan). The iCycler iQ real‐time detection system (Bio‐Rad, Hercules, CA, USA) with SYBR‐Green I (Bio‐Rad) was used to perform real‐time qPCR. Gene‐specific primers were used, and specificity for the primers was checked by melting curve analysis. Cycling conditions were 95°C for 3 min, followed by 40 cycles of 95°C for 30 s, and 60°C for 30 s. To quantify the target gene expression, each gene was normalized using GAPDH as internal control. Primer sequences are listed in Figure S18.

### 4.12 Determination of mRNA stability

10^5^ Lamin A and Progerin HEK293 cells were first cultured in 6‐well plates overnight, followed by 5 μg/ml Actinomycin D. Cell lysates were then collected at indicated times by TRIZOL. Total RNA was reverse transcribed to complementary DNA, and LPA_3_ mRNA decline was determined by real‐time qPCR. Primer sequences are listed in Figure S18.

### 4.13 Statistical analysis

Data were analyzed by one‐way analysis of variance (ANOVA) and Fisher's protected least significant difference (LSD) tests (Stat‐View, Abacus Concept, Berkeley, CA, USA). Each result was obtained after three to six independent experiments, and a *p* value of <.05 was considered statistically significant.

## CONFLICT OF INTEREST

None declared.

## AUTHOR CONTRIBUTIONS

Wei‐Min Chen, Hsinyu Lee, Benjamin Chen, and Yueh‐Chien Lin: conceived and designed the study. Hsinyu Lee and Benjamin Chen: performed as administrative support. Hsinyu Lee and Benjamin Chen: contributed to the provision of study materials or patients. Wei‐Min Chen and Jui‐Chung Chiang: collected and assembled the data. Wei‐Min Chen, Yu‐Nung Lin, Ya‐Chi Chang, Kao‐Yi Wu, and Jung‐Chien Hsieh: involved in animal study. Wei‐Min Chen, Benjamin Chen, and Hsinyu Lee: analyzed and interpreted the data. Wei‐Min Chen: wrote the manuscript. All authors involved in final approval of manuscript.
