# Supplementary material for: Lysophosphatidic acid receptor LPA3 prevents oxidative stress and cellular senescence in Hutchinson–Gilford progeria syndrome
Source: Aging Cell. 2019 Nov 12;19(1):e13064. doi: 10.1111/acel.13064 (PMC6974717; doi:10.1111/acel.13064)
Supplement: Supplementary file 1 [file ACEL-19-e13064-s001.pdf]

Supplemental protocol

Figure S19. Establishment and maintenance of LPA_3 knockout Zebrafish

LPA_3 knockout zebrafish were established using the Transcription Activator-like effector nuclease (TALEN) gene knock out system. TALEN target sites were designed by ZGene Biotech Inc (Taipei, Taiwan) according to the zebrafish LPA_3 genomic sequence (CABZ01059551.1). The left arm target site of LPA_3 is GATCATAGCAGCGGTG, and the right arm target site is TCACTACCCTTTCTACT. Each arm of the target sequence was cloned into the pZGB2 TALEN vector. 5'-capped TALEN mRNA was generated by *in vitro* transcription (mMESSAGE SP6 kit, Thermo Fisher Scientific Invitrogen) using *Not*I-linearized pZGB2 TALEN vectors as template. Equal amounts (100 ng/μl) of left and right TALEN mRNA were injected into the cytoplasm of single cell stage zebrafish embryos.

Figure S1

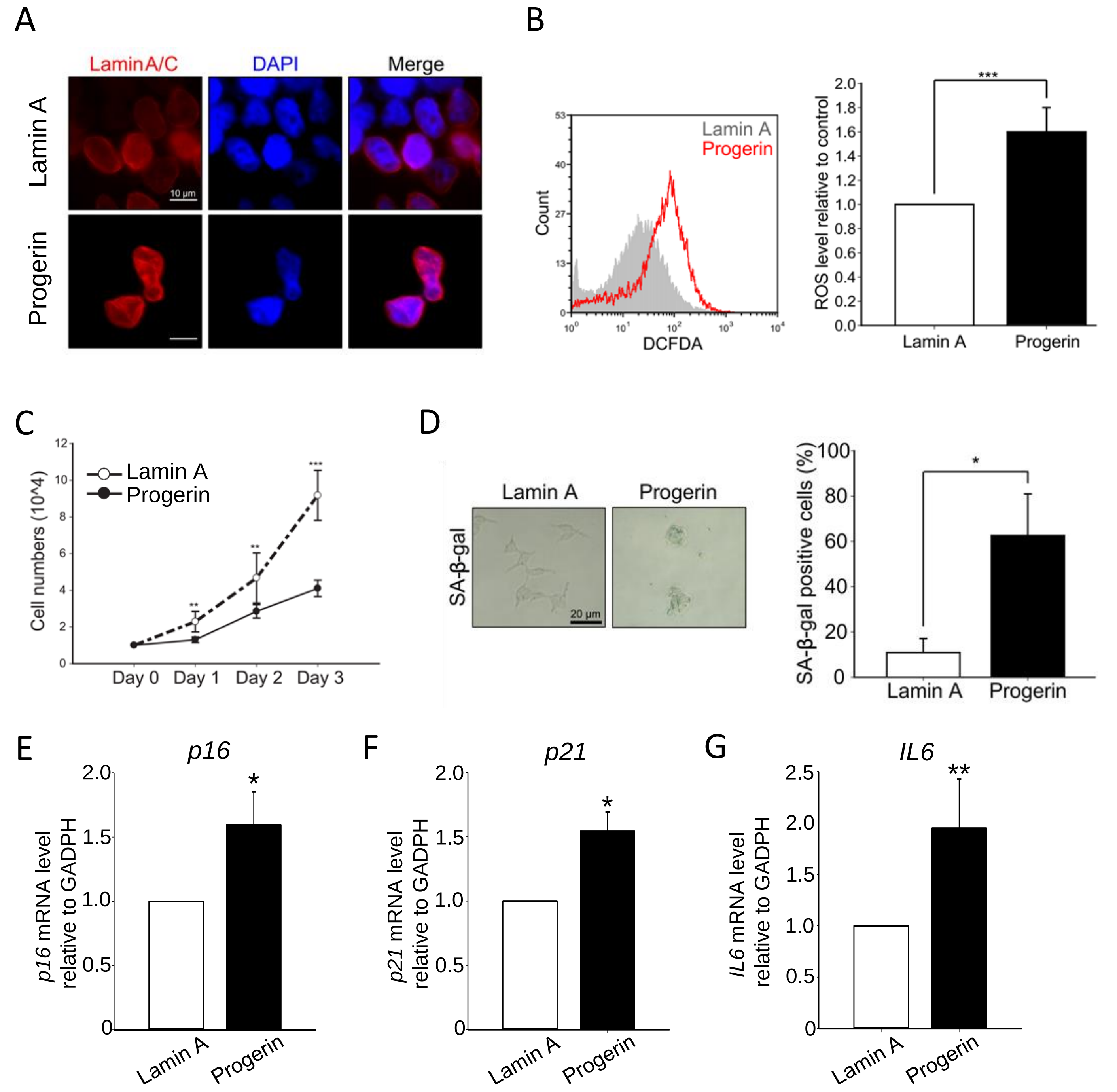

Figure S1. Expression of Progerin in HEK293 elevates ROS level, cell senescence, and retards cell proliferation. (A) Immunofluorecent staining with Lamin A/C antibody revealed abnormal nuclear shapes in Progerin HEK293 cells. (B) By flow cytometry, DCF-DA staining showed elevated ROS level in Progerin HEK293 cells. (C) Revealed by cell counting, expression of Progerin reduced HEK293 cell proliferation. (D) X-gal staining showed higher percentages of senescent cells in Progerin HEK293 cells. Quantification of β-gal positive cells were presented at the right side. (E) By real-time qPCR, expression of Progerin increased mRNA level of *p16*. (F) By real-time qPCR, expression of Progerin increased mRNA level of *p21*.(G) By real-time qPCR, expression of Progerin increased mRNA level of *IL6*. ANOVA and Student's *t*-test; *p<0.05, **p<0.01, ***p<0.001.

Figure S2

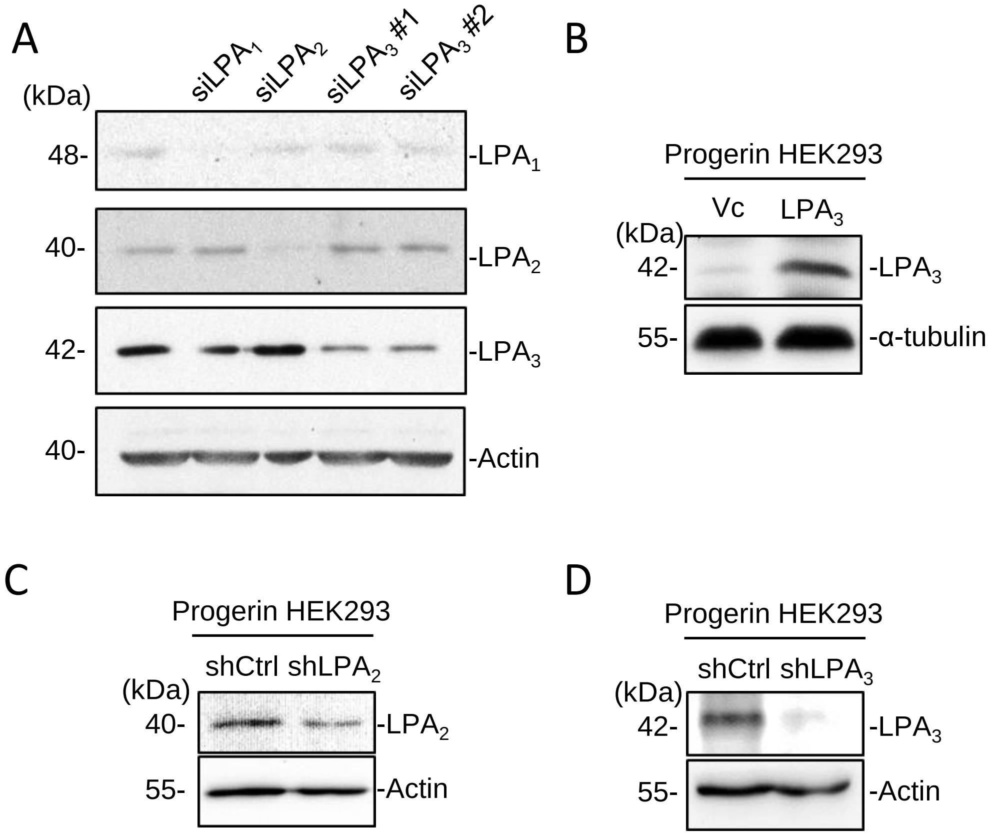

Figure S2. Manipulation of LPA receptors by RNA interference and overexpression. (A) Western blot results showed knockdown of LPA_1 by siRNA ($siLPA_1$), LPA_2 by siRNA ($siLPA_2$), and LPA_3 by two individual siRNAs ($siLPA_3$ #1 and #2). (B) Western blot with LPA_3 antibody showed the overexpression of LPA_3. (C) Western blot with LPA_2 antibody showed the knockdown of LPA_2 by $shLPA_2$. (D) Western blot with LPA_3 antibody showed the knockdown of LPA_3 by $shLPA_3$.

Figure S3

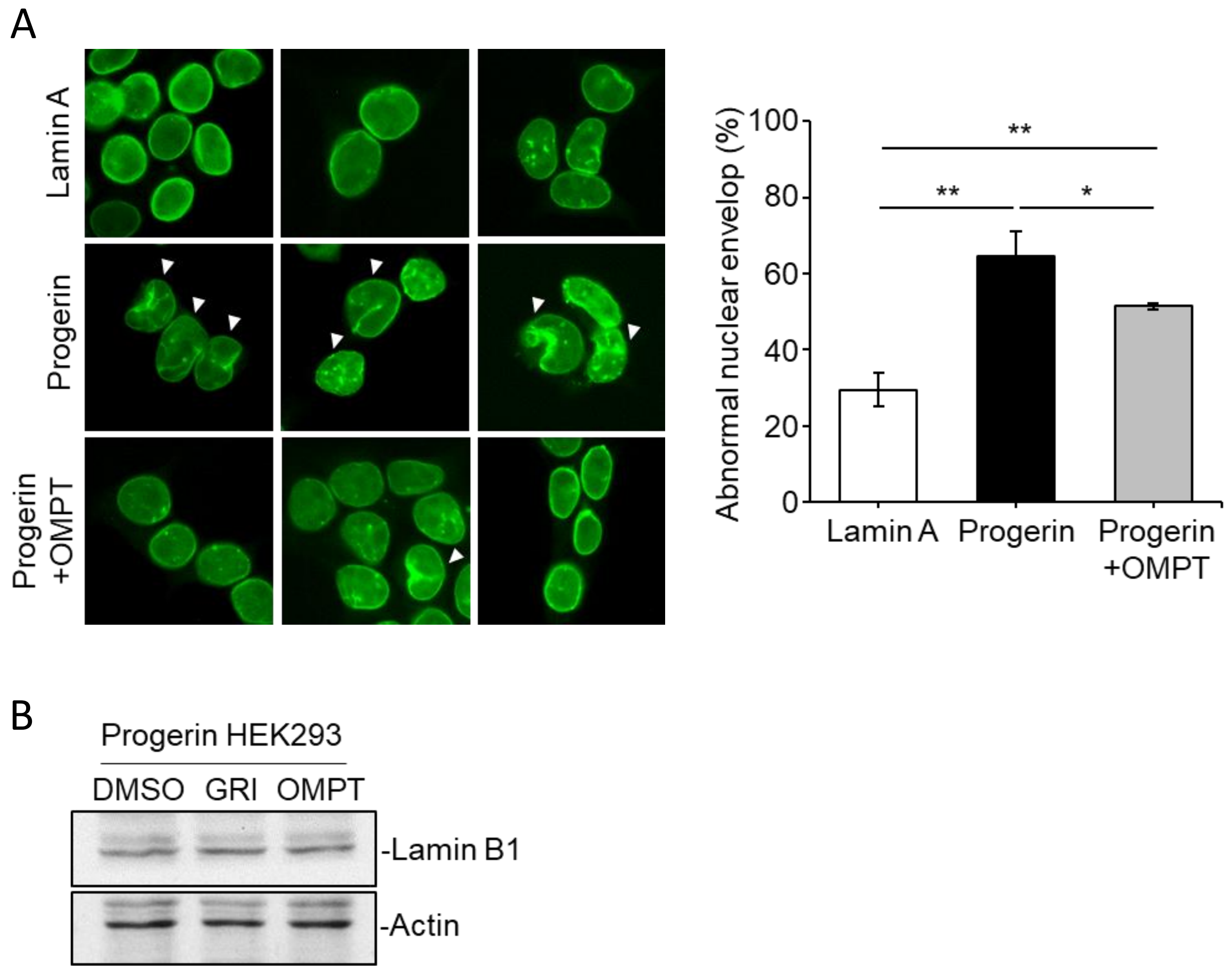

Figure S3. Activation of LPA3 ameliorates abnormity of nuclear envelop caused by Progerin, but not protein level of Lamin B1. (A) Immunofluorecent staining with Lamin A/C antibody revealed that treatment of OMPT for one week ameliorated abnormal nuclear shapes in Progerin HEK293 cells. Quantification is shown in left panel. (B) Western blot showed that treatment of OMPT for 2 days had no effects on rescuing protein level of Lamin B1 in Progerin HEK293 cells. ANOVA and Student's *t*-test; **p*<0.05, ***p*<0.01, ****p*<0.001.

Figure S4

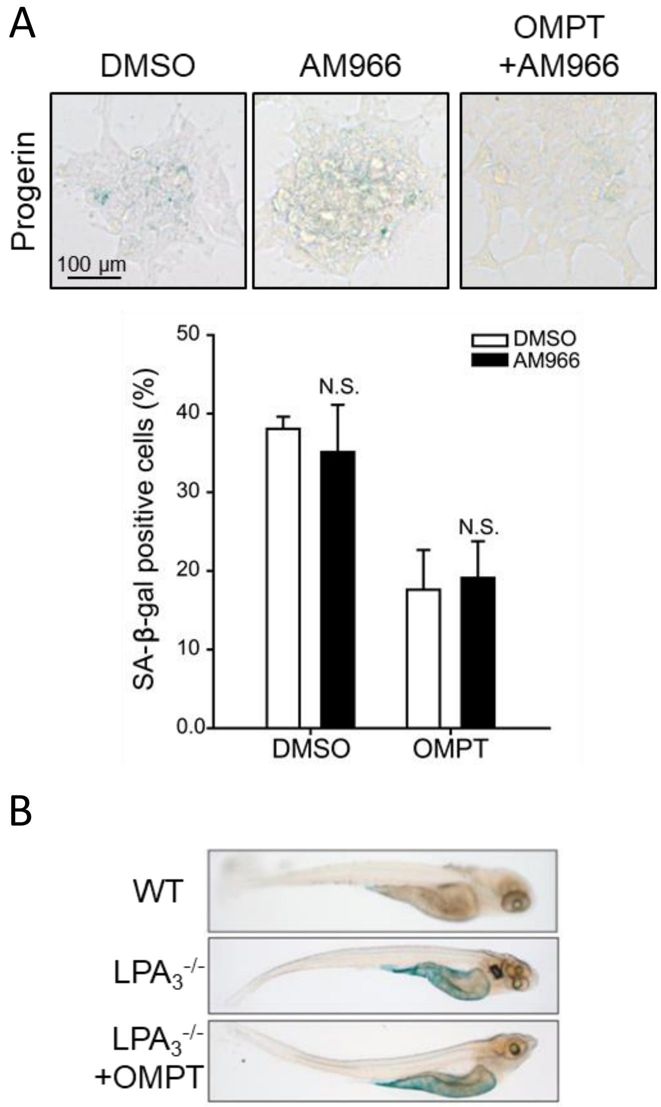

Figure S4. OMPT is shown to specifically activate LPA_3 to reduce senescence. (A) Representative images of senescence associated β-gal staining assay and its quantified results. Inhibition of LPA_1 by treatment of 100 nM AM966 for 4 days has no effects on cell senescence of Progerin HEK293 cells. In addition, co-treatment of 100 nM AM966 and 100 nM OMPT for 4days didn't abolish rescuing effects of OMPT on cell senescence of Progerin HEK293 cells. (B) $LPA_3^{-/-}$ zebrafish embryo showed high intensity of SA-β-gal staining, but couldn't be rescued by OMPT treatment. ANOVA and Student's *t*-test; *p<0.05, **p<0.01, ***p<0.001.

Figure S5

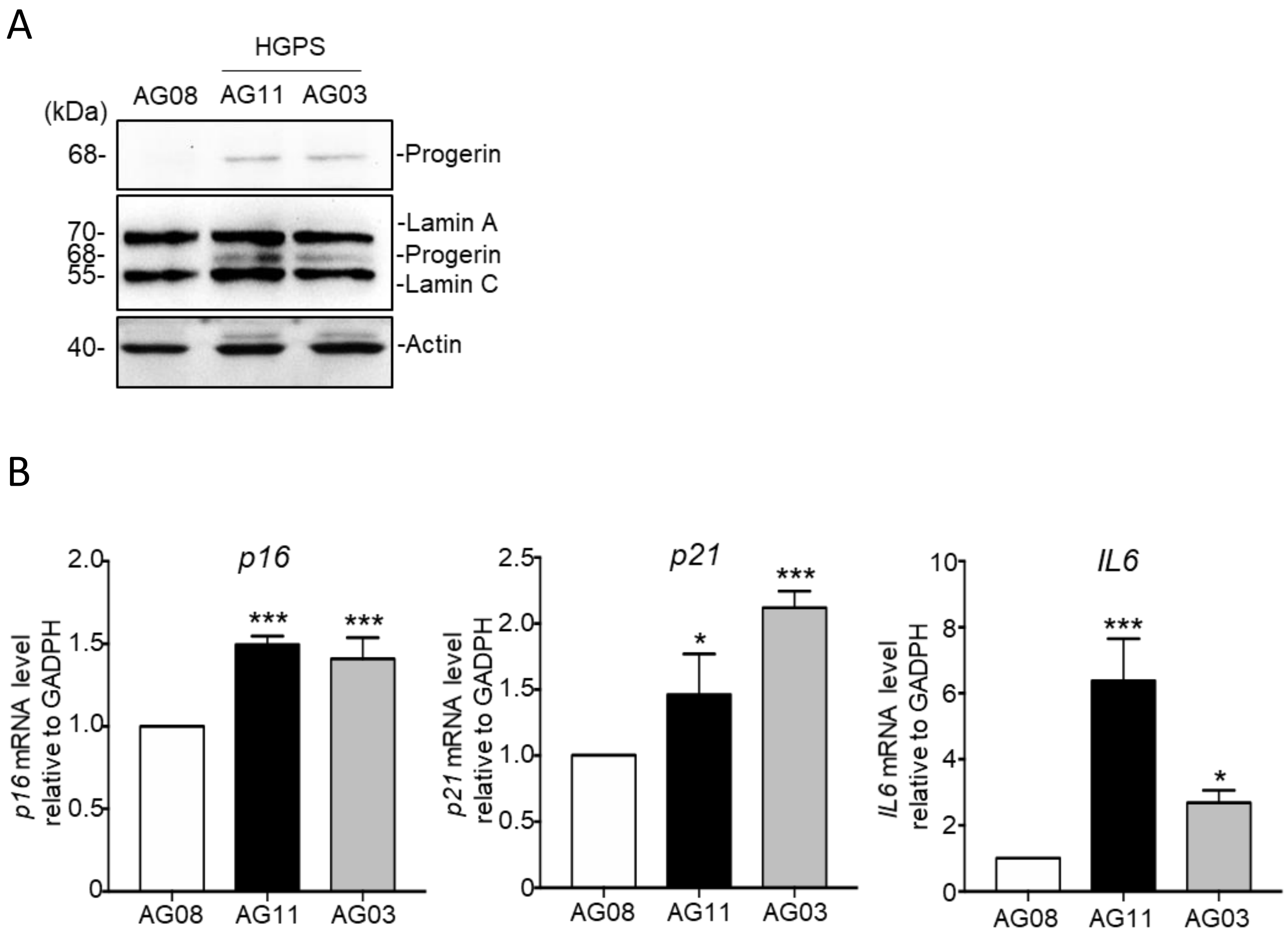

Figure S5. Senescence related genes are elevated in HGPS patient fibroblasts. (A) Immunofluorecent staining with Lamin A/C antibody and Progerin antibody revealed expression of Progerin in HGPS patient fibroblasts. (B) By real-time qPCR, expression of Progerin increased mRNA level of *p16*, *p21*, and *IL6*. ANOVA and Student's *t*-test; **p*<0.05, ***p*<0.01, ****p*<0.001.

Figure S6

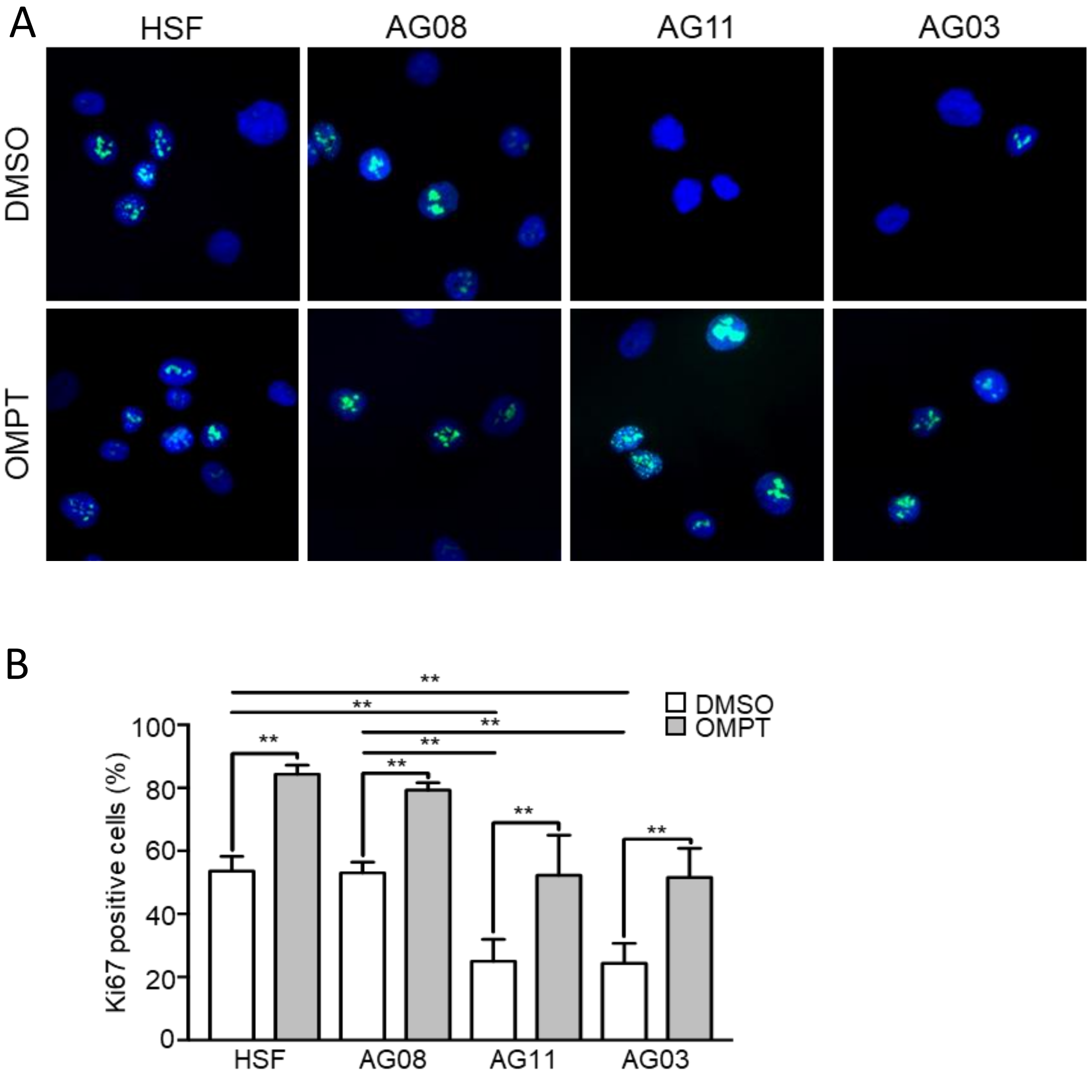

Figure S6. Activation of LPA_3 by OMPT increases Ki67 positive cells of HGPS fibroblasts. (A) Immunofluorecent staining with Ki67 antibody revealed that Progerin reduces Ki67 positive cells of HGPS patient fibroblasts. In addition, activation of LPA_3 by 100 nM OMPT for 24 hours increased Ki67 positive cells of both normal fibroblasts and HGPS patient fibroblasts. (B) Quantification of Ki67 staining results. ANOVA and Student's *t*-test; $*p<0.05$, $**p<0.01$, $***p<0.001$.

Figure S7

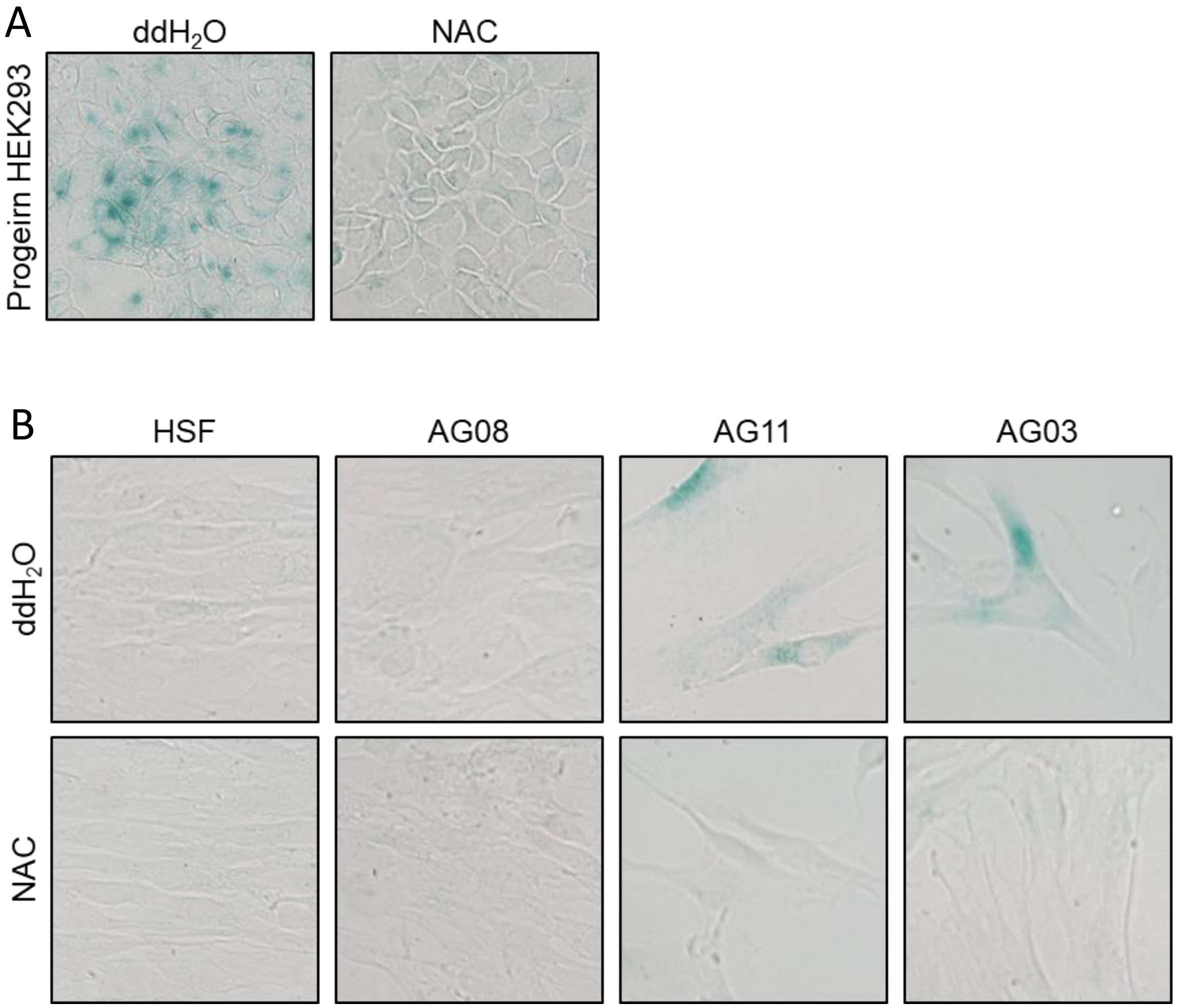

Figure S7. Treatment of NAC reduces cell senescence of Progerin HEK293 cells and HGPS patient fibroblasts. (A) Treatment of 2 mM NAC for 4 days reduced percentage of β-gal$^+$ Progerin HEK293 cells. (B) Treatment of 2 mM NAC for 4 days reduced percentage of β-gal$^+$ HGPS patient fibroblasts.

Figure S8

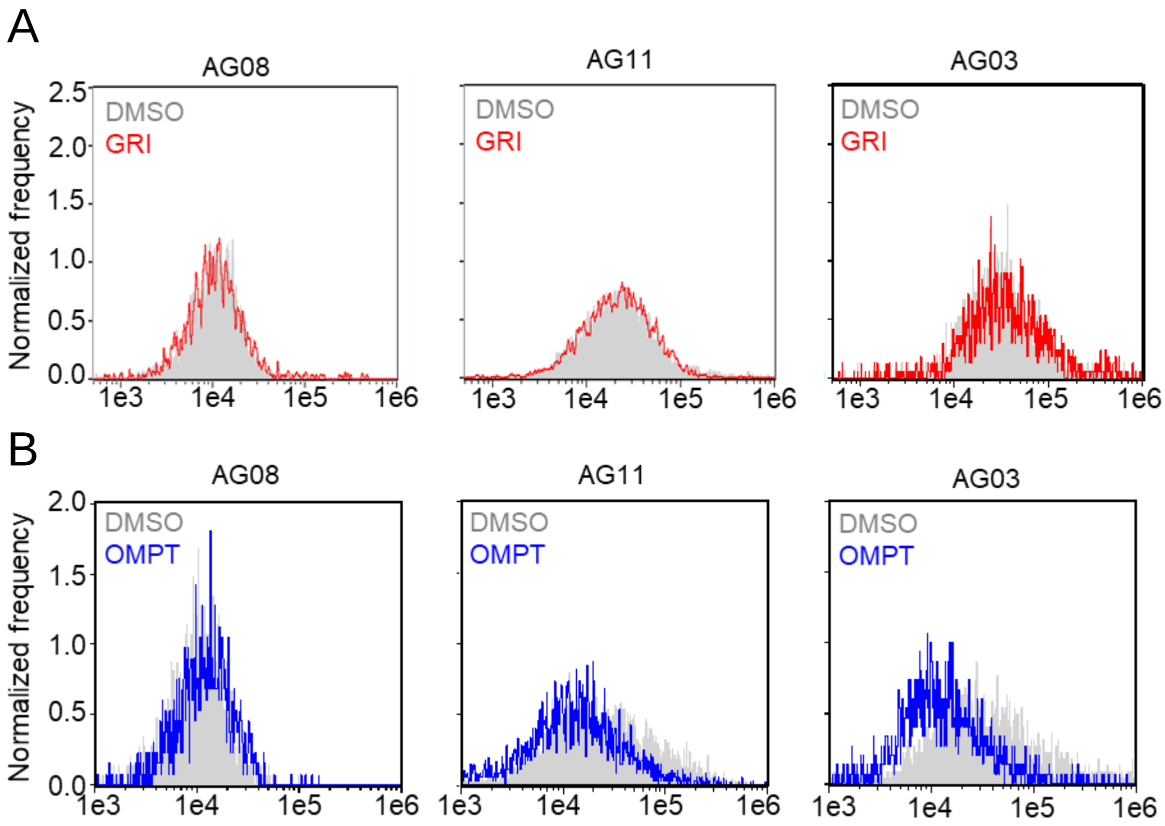

Figure S8. Corresponding images for flow cytometry results of ROS in HGPS patient fibroblasts. (A) By flow cytometry, DCF-DA staining results show that activation of LPA_2 by 5 μM GRI for 48 hours has no effects on ROS level in both HGPS AG03 and AG11 fibroblasts. (B) By flow cytometry, DCF-DA staining results show that activation of LPA_3 by 100 nM OMPT for 48 hours decreases ROS level in both HGPS AG03 and AG11 fibroblasts.

Figure S9

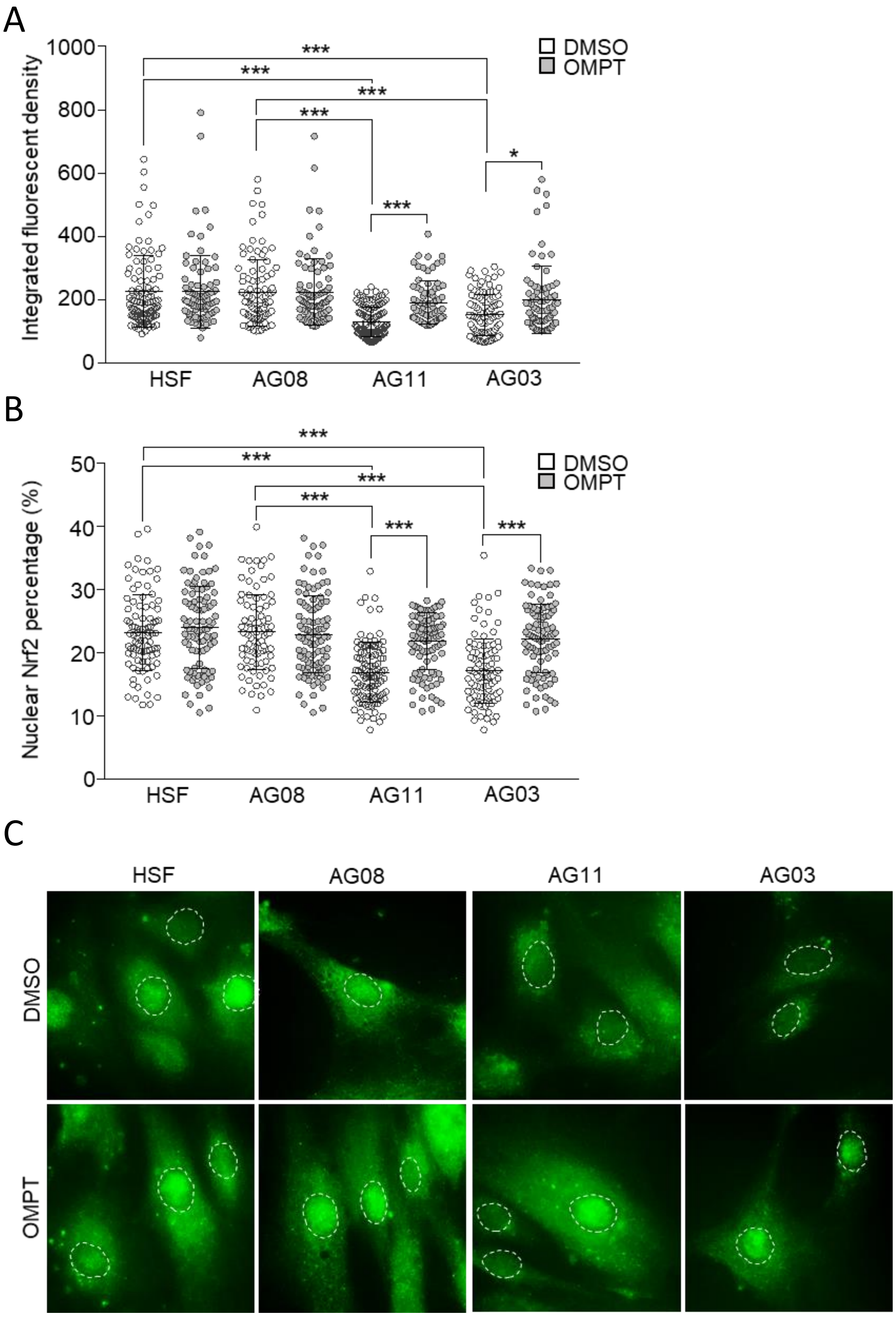

Figure S9. Activation of LPA_3 rescues expression level and translocation of Nrf2. (A) Treatment of 100 nM OMPT for 24 hours rescued protein level of Nrf2 in HGPS patient fibroblasts. (B) Treatment of 100 nM OMPT for 24 hours increased nuclear translocation of Nrf2 in HGPS patient fibroblasts. (C) Representative images for Nrf2 immuno-fluorescent images (n>100 cells).ANOVA and Student's *t*-test; *$p<0.05$, **$p<0.01$, ***$p<0.001$.

Figure S10

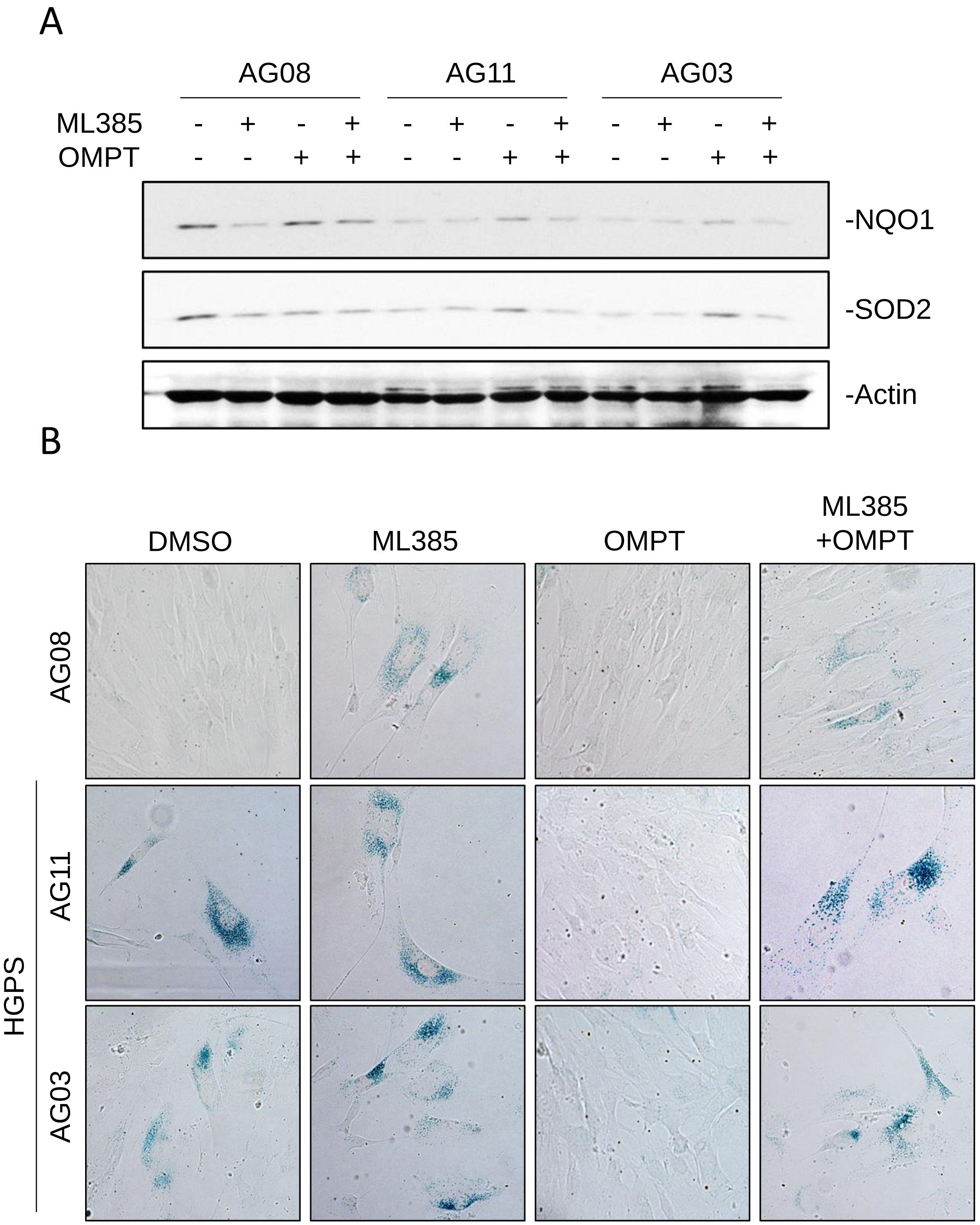

Figure S10. Nrf2 inhibitor ML385 abolishes the effect of LPA_3 on anti-oxidants and cell senescence (A) Treatment of 5 μM ML385 for 24 hours decreased protein level of anti-oxidants in AG08 cells. In addition, co-treatment of ML385 and 100 nM OMPT for 24 hours abolished rescuing effect of LPA_3 on protein level of anti-oxidants in HGPS patient fibroblasts. (B) Representative images for β-dal staining. Treatment of 5 μM ML385 for 5 days increased cell senescence of AG08 cells. In addition, co-treatment of ML385 and 100 nM OMPT for 5 days abolished rescuing effect of LPA_3 on cell senescence in HGPS patient fibroblasts.

Figure S11

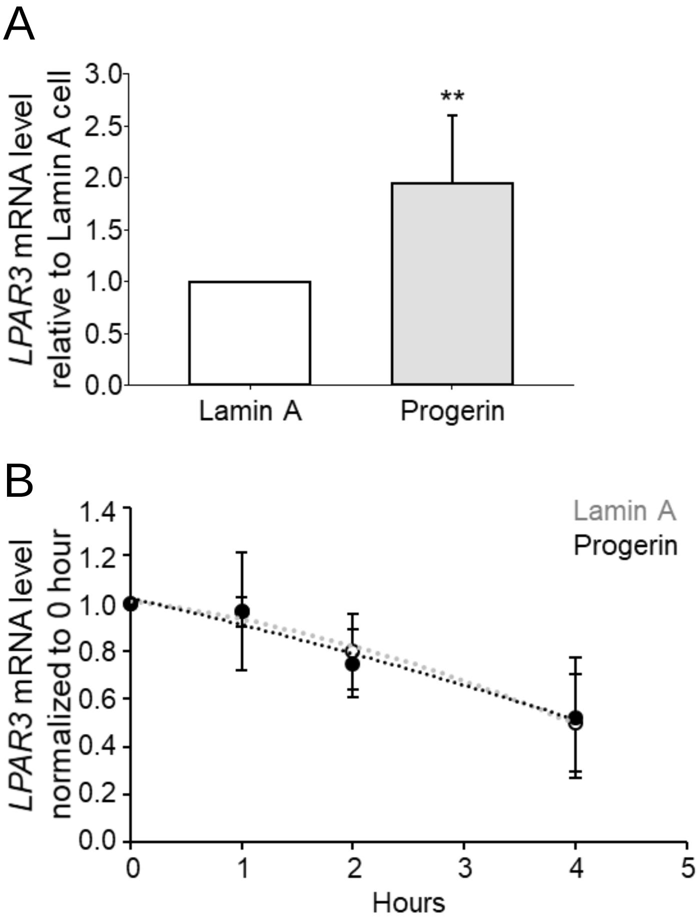

Figure S11. Decline of LPA_3 protein is not regulated by transcriptional activation nor mRNA stability in Progerin HEK293 cells. (A) Real-time qPCR analysis showed mRNA level of LPA_3 in Lamin A and Progerin HEK293 cells. (B) Real-time qPCR analysis showed decline of LPA_3 mRNA after 5 μg/ml Actinomycin D treatment to both Lamin A and Progerin HEK293 cells. ANOVA and Student's *t*-test; *$p<0.05$, **$p<0.01$, ***$p<0.001$.

Figure S12

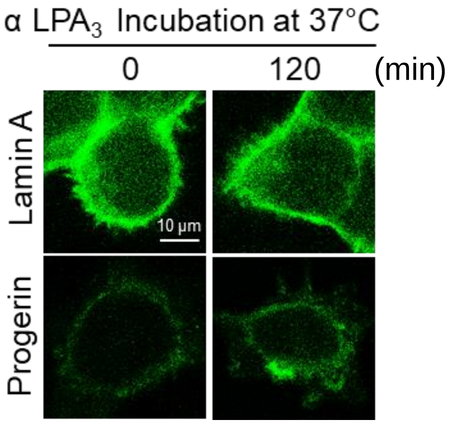

Figure S12. Internalization assay of LPA_3 without surface antibody retrieval. LPA_3 primary antibody was detected using anti-rabbit IgG conjugated with Alexa 647. At 0 minute, surface protein of LPA_3 was shown to be reduced in Progerin cells. In addition, internalization of LPA_3 was increased in Progerin cells after 120 minutes incubation.

Figure S13

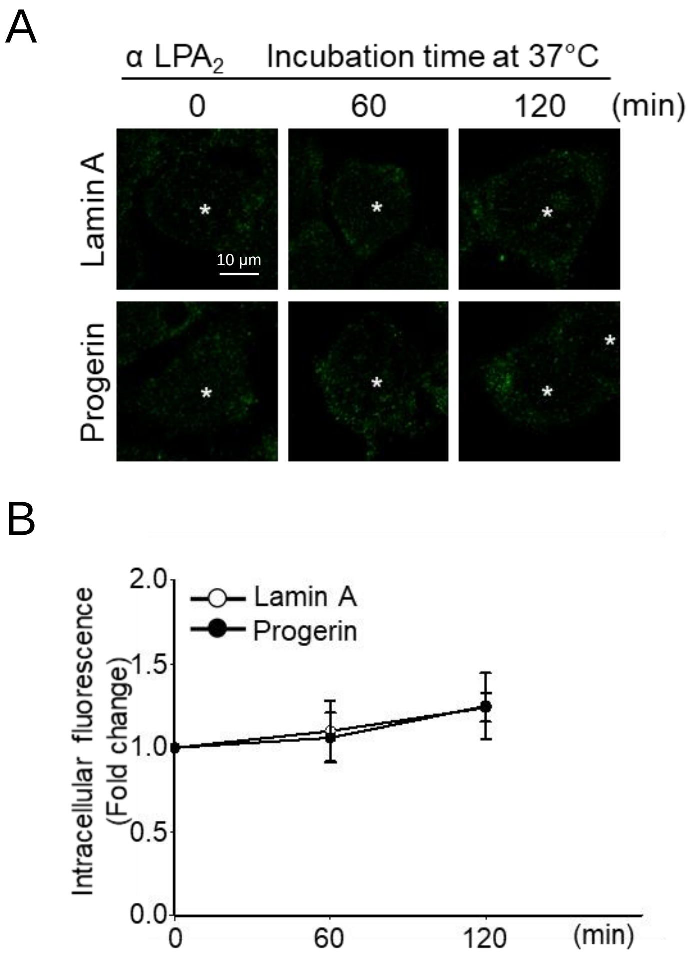

Figure S13. Internalization of LPA_2 in Progerin cells is not distinct to Lamin A cells. (A) Representative images of time-dependent LPA_2 internalization. Internalization of LPA_2 was not changed in Progerin HEK293 cells. Integrated density of Intracellular fluorescence was quantified and normalized to 0 min to indicate internalized LPA_2 in (B); $^{*}p<0.05$, $^{**}p<0.01$, $^{***}p<0.001$.

Figure S14

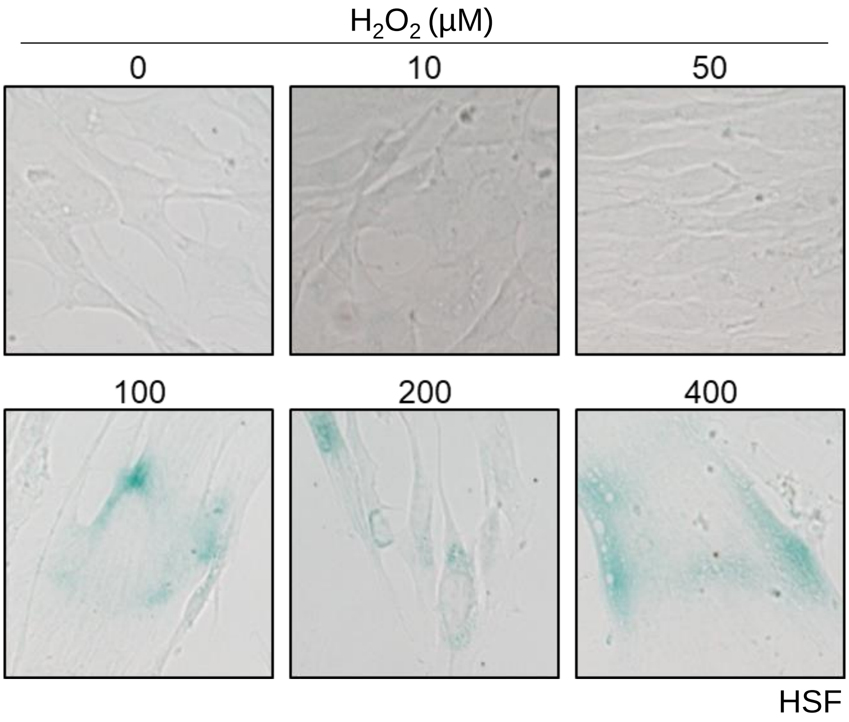

Figure S14. More than 100 µM of H_2O_2 leads to cell senescence of HSF. Treatment of 0, 10, 50, 100, 200, and 400 µM H_2O_2 to HSF for two days. More than 100 µM of H2O2, but not 10 and 50 µM, leads to cell senescence of HSF.

Figure S15

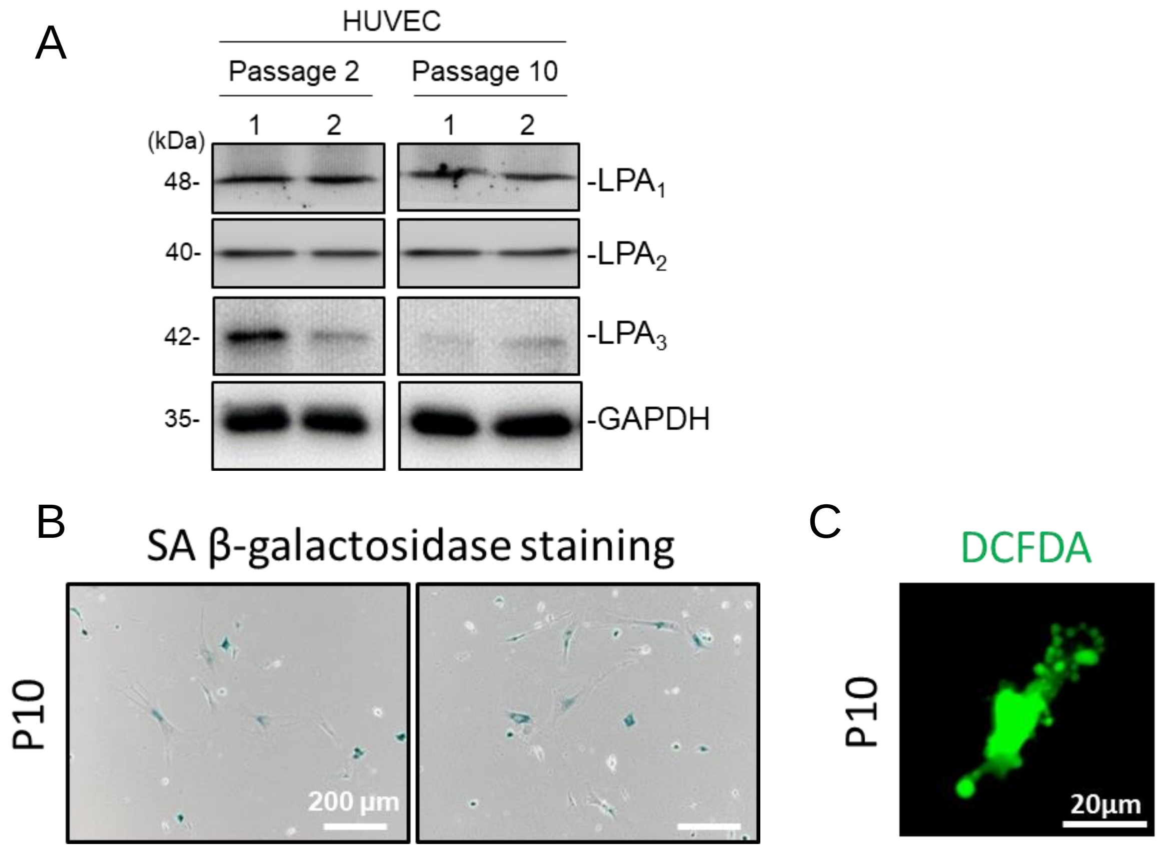

Figure S15. LPA$_3$ decreases correlates to increased ROS level and senescence in HUVEC. (A) Protein level of LPA receptors in early and late passages of HUVEC. (B) Late passage of HUVEC shows high intensity of β-gal staining. (C) late passage of HUVEC shows high level of ROS.

S.16 Western blot images developed by UVP illuminator

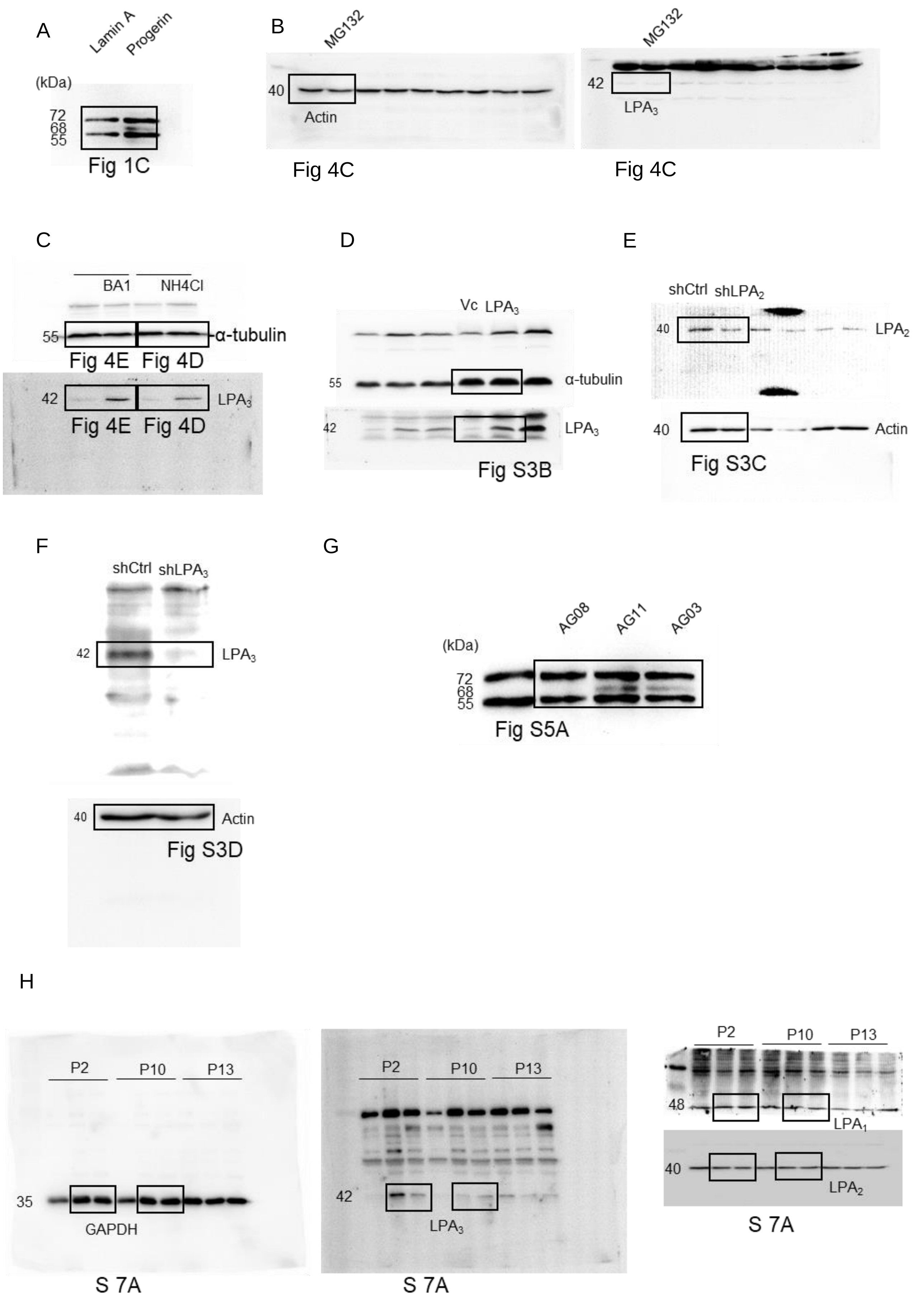

S.17 Western blot images developed by X-ray film mixer

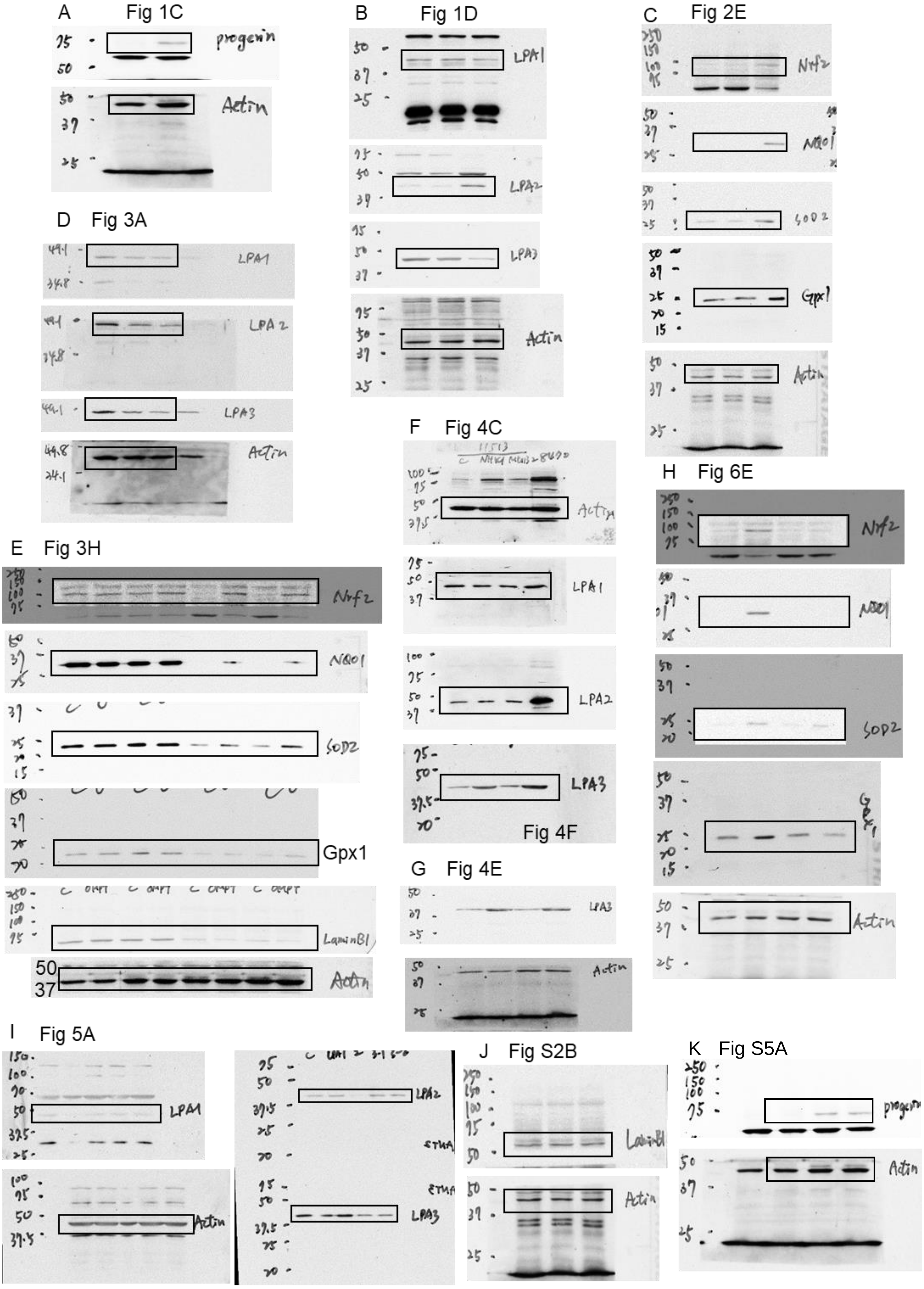

S.17 Western blot images developed by X-ray film mixer (continued)

L Fig S10A

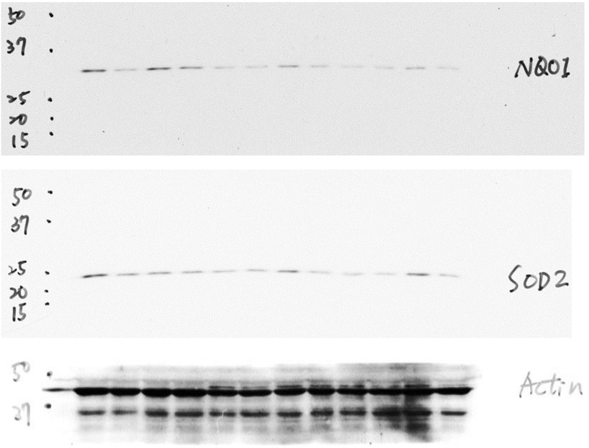

Figure. S18

Cloning

Primer	Forward 5'→3'	Reverse 5'→3'
Lamin A (EcoRI - BamHI)	CTTCGAATTCATGGAGACCCCGTCCCAGCG	GCGGATCCTTACATGATGCTGCAGTTCTGG
Progerin (1824C>T)	AGCCCAGGTGGGTGGACCCATCTCCT	AGGAGATGGGTCCACCCACCTGGGCT
Progerin 150bp deletion	GGTGGGAGCCCC*C*A*G*AACTGCAGC	GGGGCTCCCACC*T*G*G*GCTCCTGAG
LPA_2 (NheI - BamHI)	CCGCTAGCATGAATGAGTGTCACTATGACAAGCA	AAGGATCCTTAGGAAGTGCTTTTATTGCAGACTGC
LPA_3 (NheI - BamHI)	AAGCTAGCATGGTCATCATGGGCCAGTG	AAGGATCCCTAAAGGGTGGAGTCCATCAG
LPA_3 (EcoRI - BamHI)	GGGAATTCATGAATGAGTGTCACTATG	GTGGATCCTTAGGAAGTGCTTTTATTG

* phosphorothiate bond

Check and sequencing

Primer	Forward 5'→3'	Reverse 5'→3'
Lamin Cryptic splice site	TGAGTACAACCTGCGCTCGC	TTACATGATGCTGCAGTTCTGGGG
pCMV 559-584	GGTTTAGTGAACCGTCAGATCCGCTA	
LMNA 701-720	AGCGTGAGTTTGAGAGCCGG	

Realtime qPCR

Primer	Forward 5'→3'	Reverse 5'→3'
GAPDH	AAGGTGAAGGTCGGAGTC	TGTAGTTGAGGTCAATGAAGG
LPA_3	GAAGCTAATGAAGACGGTGATGA	AGCAGGAACCACCTTTTCAC
p16	GGGGGCACCAGAGGCAGT	GGTTGTGGCGGGGGCAGT
p21	TGAGCCGCGACTGTGATG	GTCTCGGTGACAAAGTCGAAGTT
IL6	CCAGCTATGAACTCCTTCTC	GCTTGTTCCTCACATCTCTC
zebrafish p16	CGAGGATGAACTGACCACAGC	CAAGAGCCAAAGGTGCGTTAC
zebrafish p21	CAAGCCAAGAAGCGTCTAGTG	AACGGTGTCGTCTCTGGTTC
zebrafish IL6	TCCTGGTGAACGACATCAAA	TCATCACGCTGGAGAAGTTG
zebrafish Gpx1a	CAGATGAACGAGCTCCACAG	CCATTCACTTCCAGCTTCTCC
zebrafish SOD2	ACTGTGTGACGGACTAGAGC	CAGATGTGAGGCTCAAGTGC
zebrafish NQO1	CGAGATGTTGCAGTTCAGGC	ATCGACCCTCTTTCCATGCA

**siRNA (target sites)**	Sense 5'→3'	Antisense 5'→3'
LPA_1 (609-629)	5'-UUGCAAUCGAGAGGCACAUUAdTdT-3'	5'-UAAUGUGCCUCUCGAUUGCAAdTdT-3'
LPA_2 (843-863)	5'-CCUGGUCAAGACUGUUGUCAUdTdT-3'	5'-AUGACAACAGUCUUGACCAGGdTdT-3'
LPA_3-1 (1376-1396)	5'-CCAUUAAUCACUGCUAGAUUUdTdT-3'	5'-AAAUCUAGCAGUGAUUAAUGGdTdT-3'
LPA_3-2 (1026-1046)	5'-CAGUACAUAGAGGAUAGUAUUdTdT-3'	5'-AAUACUAUCCUCUAUGUACUGdTdT-3'

Figure. S19

Antibody	Host	Cat. No.	Company
Lamin A/C	Rabbit	ab108595	Abcam
LPA_1	Rabbit	ab23698	Abcam
LPA_2	Rabbit	ab38322	Abcam
LPA_3	Rabbit	ab23692	Abcam
LPA_3	Mouse	sc-390270	Santa Cruz
Nrf2	Rabbit	CST-12721	Cell signaling
α-tubulin	Mouse	GT114	Genetax
Actin	Goat	sc-1616	Santa Cruz
SOD2	Rabbit	CST-13141	Cell signaling
Gpx1	Rabbit	CST-3286	Cell signaling
NQO1	Mouse	CST-3187	Cell signaling
GAPDH	Rabbit	GTX100118	Genetax
Progerin	Mouse	Sc-81611	Santa Cruz
Ki67	Rabbit	CST-9027	Cell signaling
